# The impact of encouraging utterances by a human partner and a conversational robot on older adults’ subjective well-being: a 6-week sustained interaction

**DOI:** 10.3389/frobt.2026.1866995

**Published:** 2026-10-09

**Authors:** Lingxuan Xiang, Hideaki Kikuchi

**Affiliations:** 1 Graduate School of Human Sciences, Waseda University, Saitama, Japan; 2 Faculty of Human Sciences, Waseda University, Saitama, Japan

**Keywords:** emotional support, encouragement strategies, human–robot interaction, long-term interaction, older adults, social robots, well-being

## Abstract

**Introduction:**

In human–human communication, encouragement has been closely associated with subjective well-being (SWB). However, the long-term effects of encouragement in human–robot interaction remain unclear, particularly in comparison with human-delivered encouragement and across different encouragement strategy types. SWB comprises both affective well-being (AWB) and cognitive well-being (CWB). This study investigated whether sustained verbal encouragement from a human partner and a conversational robot could enhance older adults’ SWB.

**Methods:**

A two-phase design was adopted. First, a preliminary online survey with 55 older Japanese adults (aged 65–74) was conducted to select encouragement strategies for the main experiment, identifying offering specific actions (high-efficacy) and distraction (low-efficacy) as two encouragement strategies that differed significantly in participants’ perceived effectiveness (p < 0.05). Based on this survey, 18 older Japanese adults (aged 62–90) were recruited for an exploratory 6-week longitudinal experiment. Participants interacted with either a human partner or a conversational robot once per week and were randomly assigned to one of four conditions: human partner or social robot (RoBoHoN) crossed with high- or low-perceived-efficacy encouragement strategy. SWB was assessed using questionnaires, while post-session open-ended responses, diary entries, and post-experiment interviews provided qualitative data on encouragement experience, perceived SWB changes, and participants’ perceived sources of SWB change.

**Results:**

Results suggested that high-efficacy verbal encouragement may positively influence older adults’ SWB, possibly with a stronger effect than low-efficacy encouragement, with a crossover-like pattern emerging: for AWB, the advantage of high-efficacy over low-efficacy encouragement tended to be more pronounced in the robot condition (Δ gap = 2.8) than the human condition (Δ gap = 0.5), whereas for CWB, it appeared to be more pronounced in the human condition (Δ gap = 2.0) than the robot condition (Δ gap = 0.2); these crossover trends should be interpreted with caution given the small sample size. Qualitative findings further suggested that the conversation itself may have contributed to participants’ perceived emotional support in the Japanese cultural context.

**Discussion:**

These preliminary longitudinal findings suggest that social robots and human partners may both contribute to supporting older adults’ SWB and may play complementary roles.

## 1 Introduction

The global population is aging at an accelerating pace. By 2050, the global population aged 65+ is projected to reach 1.6 billion (United Nations Department of Economic and Social AffairsPopulation Division, 2024). Japan is among the most aged countries, with older adults already comprising 29.3% of its population (Cabinet Office of Japan, 2025). Aging brings daily challenges for older adults, including physical decline, chronic pain, and loneliness. These concerns are not clinical disorders but recurrent daily hassles (Kanner et al., 1981). Providing sustained, low-threshold emotional support for such everyday worries has become an important social need in an aging society. In this context, chatbots and social robots capable of natural conversation are increasingly being considered as potential resources for providing emotional support. However, whether these systems can provide long-term emotional support of a quality comparable to that of human interaction partners remains unclear.

In supportive communication research, attentive listening is widely recognized as an effective behavior for alleviating immediate stress (Weger et al., 2014). However, the cognitive reappraisal theory of social support suggests that effective supportive communication depends more critically on helping recipients achieve cognitive reappraisal (Burleson and Goldsmith, 1998). Encouragement is a more active form of supportive behavior than passive listening (Pauw et al., 2018). Encouragement not only addresses immediate emotions but may also promote long-term subjective well-being (SWB) by reframing concerns, enhancing perceived coping ability, and sustaining motivation (Burleson and Goldsmith, 1998; Khan, 2013). More broadly, social support has been shown to promote psychological well-being (Cohen and Wills, 1985), and encouragement from family and educators is significantly associated with life satisfaction and well-being (Edwards and Lopez, 2006; Khan, 2013). Furthermore, different encouragement strategies may operate through distinct psychological mechanisms, and their effectiveness may vary depending on strategy type (Ogawa, 2014). However, this evidence is entirely based on human-human interaction. Whether these strategy differences hold for robot-delivered encouragement is unclear.

In human-robot interaction (HRI), supportive interaction research has focused on companionship, listening, reminders, or general conversation (Spitale et al., 2025; Matheus et al., 2025). Attention to encouragement as a more active form of support remains limited, and systematic longitudinal comparisons of different encouragement strategies are rare. Existing studies have shown that users are more likely to interpret human-delivered encouragement as genuine care, whereas encouragement from a social robot may be perceived as a system output. This attributional difference may affect the perceived credibility of the encouragement (Nass and Moon, 2000; Lucas et al., 2014), and may in turn influence its perceived effectiveness. At the same time, the embodiment of social robots may reduce social evaluation pressure, providing older adults with a low-burden space for emotional disclosure that is difficult to access in interpersonal relationships (Lucas et al., 2014; Holthöwer and van Doorn, 2023). Therefore, directly comparing the long-term effects of encouragement delivered by a human partner versus a social robot under identical strategy conditions is of great significance for understanding the theoretical boundaries of supportive interaction in HRI and for designing support systems that truly meet the needs of older adults. Moreover, if social robots can provide low-burden, sustainable daily emotional support without displacing meaningful human relationships, they hold considerable promise for both HRI research and well-being promotion in aging societies.

Based on the above background, this study investigates whether social robot-delivered encouragement and human-delivered verbal encouragement are associated with different longitudinal changes in older adults’ SWB, and whether these changes vary by encouragement strategy efficacy. The study has two phases. First, a preliminary survey with 55 older adults identified high- and low-efficacy encouragement strategies. Then, an exploratory 6-week longitudinal experiment with 18 older adults was conducted, in which participants were randomly assigned to four conditions (human partner or social robot crossed with high- or low-efficacy strategy) and engaged in weekly structured conversations, with SWB assessed at multiple time points.

This study makes three primary contributions. First, to the best of our knowledge, this is the first longitudinal study to directly compare the long-term effects of encouragement on SWB between a social robot and a human partner among older adults. Second, the mixed-methods design integrates post-dialogue ratings, diaries, and attribution interviews. This allows us to account for the effects of external life events when examining the conversational intervention’s impact on SWB change, and to explore the psychological mechanisms underlying the differential effects of high- and low-efficacy strategies across human-human and human-robot interaction contexts. Third, this study examines older adults’ patterns of emotional disclosure in the Japanese cultural context, where social norms around not burdening others may discourage everyday sharing, offering potential practical implications for designing support systems tailored to older Japanese adults.

This study addresses two research questions.

RQ1: How do older adults’ SWB changes differ over 6 weeks of sustained interaction when high- and low-efficacy encouragement strategies are delivered by a social robot versus a human partner?
RQ2: Does the perceived effectiveness of high-versus low-efficacy encouragement strategies change over time, and does it differ when delivered by a social robot versus a human partner?

## 2 Related work

### 2.1 Definition and measurement of subjective well-being

Subjective well-being (SWB) refers to an individual’s overall subjective evaluation of their quality of life. SWB comprises two dimensions: affective well-being (AWB), reflecting daily emotional balance, and cognitive well-being (CWB), reflecting evaluation of life circumstances (Diener et al., 2003). Common methods include self-report questionnaires, diaries, and experience sampling (ESM). Each has trade-offs: questionnaires are convenient but subject to recall bias (Squires et al., 2011); diaries risk inconsistent compliance (Squires et al., 2011); and ESM is precise but may disrupt daily life (Stieger and Reips, 2019). This study adopted a combination of questionnaires and diaries to capture different SWB components and cumulative changes over time. For the specific measures, AWB was assessed using the WHO-5 Well-Being Index, extensively validated in older adult populations (Topp et al., 2015). Life satisfaction, as an indicator of CWB, was measured using the Satisfaction With Life Scale (SWLS), widely used in longitudinal studies to capture sustained cognitive changes (Diener et al., 1985). The distinction between AWB and CWB broadly parallels the conceptual differentiation between hedonic and eudaimonic well-being in the broader well-being literature (Ryan and Deci, 2001): AWB corresponds to hedonic daily emotional experience, and CWB to eudaimonic cognitive life evaluation.

### 2.2 Definition and classification of encouragement

Encouragement refers to supportive verbal expressions aimed at alleviating negative emotions such as sadness, anxiety, dissatisfaction, or regret (Tanaka, 2015). Following Tanaka (2015), encouragement can be classified into five types: reassurance and affirmation (Type A), expressing concern (Type B), encouragement to take action (Type C), offering specific actions (Type D), and distraction (Type E). The definitions and representative examples for each type are presented in Table 1. This classification provides an operational framework for designing and comparing encouragement strategies in this study. Encouragement may play a unique role in HRI, but its effects have not been systematically examined.

**TABLE 1:** Classification of encouragement types and examples.

Encouragement type	Definition	Examples
Type A: Reassurance and Affirmation	Affirm the other person’s situation or future with positive praise or reassurance	“It happens to everyone, so don't worry about it.” “You are amazing! I’m sure you can do it.”
Type B: Expressing Concern	Show sympathy or understanding by asking questions, indicating genuine concern	“That sounds tough. Are you okay?” “I see. You’re not pushing yourself too hard, are you?”
Type C: Encouragement to Take Action	Motivate the person to act on their issues, though this can sometimes feel burdensome	“Why not give it a try first? It might help to experiment a little!” “It’s more enjoyable if you think of it lightly. Don’t take it so seriously.”
Type D: Offering Specific Actions	Offer help, emphasizing assistance rather than doing it together	“If there’s anything I can do, I’ll help you out.” “I’ll look that up for you and let you know.”
Type E: Distraction	Shift the person’s focus away from their problems to relieve stress. Suggestions should be light and non-pressuring	“How about soaking in a nice warm bath?” “How about having a delicious dessert?”

The five-type classification and definitions are based on Tanaka (2015). The example phrases were adapted from Tanaka (2015) and supplemented by the authors, and were reviewed by three native Japanese speakers to confirm their correspondence with each type. The stimuli used in the preliminary survey were constructed on the basis of these examples.

### 2.3 Social robots for well-being

The effects of social robots on mental health and well-being have been widely examined. In single or short-term interactions, brief engagement with Paro reduced psychophysiological stress (Aminuddin et al., 2016); expressing and receiving gratitude through a voice agent enhanced positive affect and SWB (Furukawa et al., 2020); and social robots reduced children’s vaccination anxiety through conversational strategies such as casual chat, questioning, and distraction (Rossi et al., 2020). In repeated or long-term interactions, a 5-week study with ten sessions found that adults interacting with Pepper showed increased self-disclosure, improved emotional states, reduced loneliness, and enhanced perceptions of social presence (Laban et al., 2024). A subsequent study with informal caregivers found similar results, including improved cognitive reappraisal of their caregiving role (Laban et al., 2025). Together, these findings suggest that the effects of social robots may vary across interaction duration and context.

In older adults, social robot interventions may positively affect depression, loneliness, and psychological well-being. A meta-analysis found that embodied social robot interventions significantly reduced depression and loneliness in long-term care residents (Yen et al., 2024). In Japan, a 4-week trial with BOCCO emo reduced loneliness and improved psychological well-being in older adults living alone (Murayama and Takase, 2025). Beyond examining whether such interventions improve well-being outcomes, prior research has also investigated how robots structure and mediate supportive interaction. SAR-Connect integrates a humanoid robot with a virtual reality-based activity platform to provide multidomain activities incorporating physical, cognitive, and social stimuli, and a laboratory-based study showed that the system engaged older adults in robot-mediated tasks while fostering interaction between them (Fan et al., 2021a). In a related field study, Ro-Tri used robot-mediated triadic interactions to foster social interaction between pairs of older adults in long-term care (Fan et al., 2021b). Evidence from longer deployments further suggests that the persistence of measured outcomes and users’ experiences of long-term robot use should be considered as distinct but complementary issues. A 6-month Hyodol intervention showed that effects emerged at 3 months but were no longer evident at 6 months (Lee et al., 2023), consistent with concerns that early positive outcomes in longitudinal HRI may partly reflect novelty effects (Matheus et al., 2025). A separate qualitative study of the same companion robot found that older adults who had kept Hyodol for an average of 18 months described how it had become incorporated into their everyday routines and relationships, portraying it as a health coach that reminded them to take medication and exercise, as a source of pleasure and companionship, and, when other social support was limited, as a surrogate family member (Lee et al., 2024). These findings suggest that the longer-term contribution of social robots may need to be considered not only in terms of sustained changes in standardized outcome measures, but also in terms of the interactional and relational roles that robots come to occupy in older adults’ everyday lives.

Beyond emotional support, conversational systems have also been examined as agents of behavior change. A mapping study of health chatbots identified personalization as a key element of chatbot design (Pereira and Díaz, 2019). A scoping review of health-related conversational agents found that the most frequently employed behavior change techniques concerned health education, self-management, and emotional social support (Martinengo et al., 2022). Similar approaches have also been implemented on physically embodied social robots: a fully autonomous dialogue system for behavior change coaching was developed and evaluated with the tabletop social robot Haru (Jelínek et al., 2024). Alongside these system-level implementations, other work has looked at what such interventions require in order to be personalized: the cognitive capabilities a social robot needs to support personalized behavior change interventions (Beraldo et al., 2025), and a conceptual framework linking behavior change models, user personalization, and persuasive language generation for conversational agents and social robots in healthcare (Maalouly et al., 2026). Together, these studies indicate that fostering behavior change through conversational agents and social robots requires not only system implementation but also cognitive capabilities and design frameworks that support personalization. However, this line of research has primarily focused on behavioral outcomes, such as adherence, physical activity, or lifestyle modification, with emotional support serving those outcomes rather than the affective and cognitive dimensions of well-being. In addition, personalization has primarily been discussed at the level of system architecture rather than in terms of which verbal strategies prove effective, and how their effects unfold over sustained interaction.

Overall, existing research has focused on tactile interaction, companionship, guided self-disclosure, reminders, general conversation, and robot-mediated activity and social engagement, or on structured coaching aimed at behavioral outcomes. Encouragement as a proactive, strategically differentiated form of verbal support for subjective well-being has not been systematically examined. Furthermore, many studies have not included a human interaction partner as a direct comparison condition, leaving the relative effectiveness of robot-delivered versus human-delivered support unclear.

### 2.4 Human vs. conversational agent: longitudinal comparative evidence

In HRI, there is currently no unified standard for defining “long-term interaction.” Existing studies propose at least five sessions (Kory-Westlund et al., 2022) or at least three sessions conducted over three consecutive days (Matheus et al., 2025) as reference criteria. According to these criteria, the 6-week, six-session design adopted in this study can be considered a form of long-term interaction.

Existing research has addressed two questions: how human-agent relationships develop over time, and whether agent support can match human partner effects. Regarding relationship development, a 12-week study with 25 Replika users found that human-chatbot relationships deepened over time, with trust and self-disclosure increasing along a trajectory consistent with social penetration theory (Skjuve et al., 2022). A 1-month experiment with 22 adults interacting with an LLM companion found that users actively negotiated the agent’s role and boundaries over time (Yun et al., 2026), suggesting that long-term human-agent relationships are dynamically evolving.

In addition to relationship development, another key question is whether conversational agents can match human partners’ effectiveness. A 2-week experiment with 296 first-year university students found that chatbot interaction produced smaller improvements in loneliness and positive affect than human peer interaction (Li et al., 2026). This suggests that even a chatbot specifically designed to be highly supportive may not match the effectiveness of human interaction. However, these studies used mostly young or general adults and virtual agents; whether findings generalize to older adults using social robots remains unclear.

Although social robot research with older adults has provided substantial longitudinal evidence (see Section 2.3), the focus has been on companionship, reminders, or general interaction, with little attention to encouragement as an independent strategy; moreover, these studies typically lack a human comparison condition. Existing evidence suggests that embodiment may alter anthropomorphic perceptions, engagement, disclosure, and interaction pressure (Li, 2015; Deng et al., 2019; Lucas et al., 2014; Holthöwer and van Doorn, 2023). Thus, findings from virtual agent studies may not generalize to social robot contexts, especially for older adults.

### 2.5 Research gaps

In summary, two interconnected gaps remain. First, although human-chatbot comparisons have emerged (Li et al., 2026), longitudinal studies directly comparing the long-term effects of identical encouragement strategies delivered by an embodied social robot versus a human partner in older adults remain scarce. Second, existing HRI research has largely focused on loneliness, depression, or composite measures, with limited attention to (1) the distinction between the affective and cognitive dimensions of SWB, and (2) how the perceived effectiveness of different encouragement strategies evolves over sustained interaction and the psychological mechanisms underlying such differences.

This exploratory study addresses these gaps by directly comparing longitudinal changes in older adults’ SWB associated with high-versus low-efficacy encouragement strategies delivered by a social robot versus a human partner, and by using a mixed-methods design to explore possible mechanisms through participants’ experiences and perceived sources of change, thereby providing preliminary findings on how encouragement functions over sustained interaction.

## 3 Materials and methods

### 3.1 Study overview and design

This study adopted a two-phase sequential design. Figure 1 provides an overview of the experimental design. In Phase 1 (preliminary survey), an online survey with 55 older Japanese adults identified high- and low-efficacy encouragement strategies. In Phase 2 (main experiment), 18 older Japanese adults participated in a 6-week longitudinal experiment with a 2 (partner: human vs. robot) × 2 (strategy: high-efficacy vs. low-efficacy) between-subjects design. Participants engaged in weekly structured conversations and completed SWB assessments at four time points. Methodologically, this design directly compares identical encouragement strategies delivered by a human partner versus a social robot, and employs a mixed-method approach combining SWB questionnaires, perceived encouragement effectiveness ratings, interviews, and diary entries to examine participants’ experiences and perceived sources of change alongside the quantitative trajectories.

**FIGURE 1:**
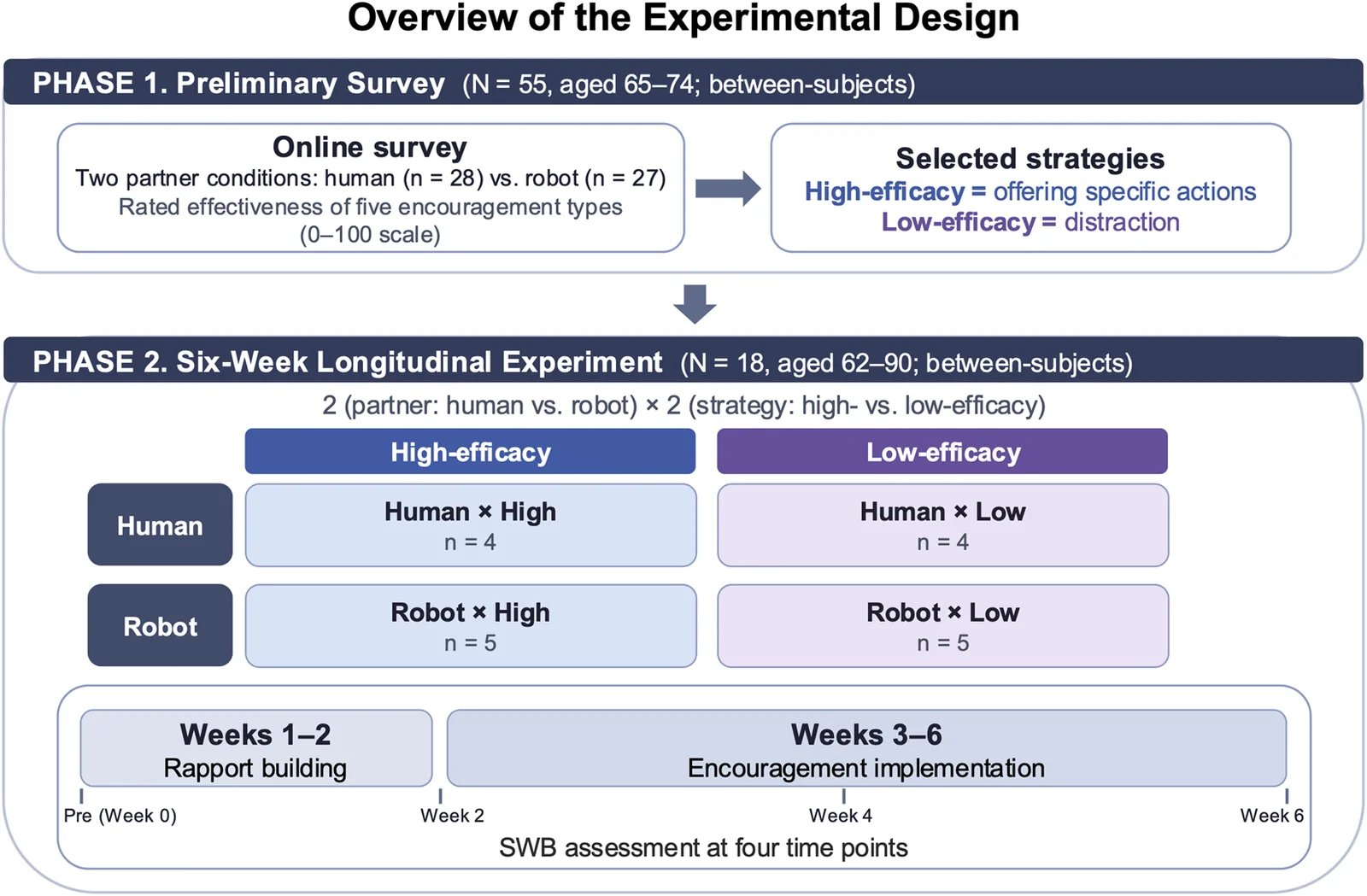
Overview of the experimental design.

### 3.2 Ethical statement

This study was approved by the Ethics Review Committee for Research Involving Human Subjects at Waseda University (Approval No. 2024-603). All participants were fully informed about the study prior to participation and provided written informed consent. Participants were informed they could withdraw at any time without consequence. All data were anonymized and used solely for academic research. All sessions were audio- and video-recorded; participants could specify whether recordings could be used for dissemination and to what extent. Data will be retained for the required period and then securely destroyed. Given the older adult population, mental health and cognitive screening were conducted at recruitment to ensure safety (see Section 3.4.2).

### 3.3 Preliminary survey

An online preliminary survey was conducted to identify encouragement strategies for the main experiment. A total of 55 older Japanese adults aged 65–74 were included (human condition: n = 28; robot condition: n = 27; overall age: M = 67.3, SD = 2.53). Participants imagined disclosing an everyday worry to a familiar conversational partner and receiving encouragement. In the human condition, the partner was described as someone close; in the robot condition, as a robot. Participants rated the perceived effectiveness of five encouragement types (see Section 2.2) on a 0–100 scale (0 = not at all encouraged, 100 = very strongly encouraged), separately for physical and psychological daily worries. Ratings were standardized within participants and analyzed using linear mixed-effects models, with Type III ANOVA and Satterthwaite’s approximation.

Results are presented in Table 2. Offering specific actions ranked highest in the human condition and was significantly higher than distraction for psychological worries in the robot condition (p < 0.05), while distraction ranked lowest across most conditions. Based on these findings, offering specific actions was selected as the high-efficacy strategy and distraction as the low-efficacy strategy for the main experiment.

**TABLE2:** Pairwise comparisons and estimated marginal mean rankings of five encouragement types by worry category and partner type.

Worry type	Partner	Significant pairs	EMM ranking
Overall	Human	D > A*, D > B**, D > E***	D > B > C > A > E
Overall	Robot	A > E**	A > B > D > C > E
Physical	Human	D > B**, D > E**	D > B > C ≈ A > E
Physical	Robot	—	A > B > E > D > C
Psychological	Human	D > E**	D > B > C > A > E
Psychological	Robot	D > E*	D > A > B > C > E

Significance codes:

*** p < .001

** p < .01

* p < .05.

A: reassurance and affirmation, B: expressing concern, C: encouragement to take action, D: offering specific actions, E: distraction.

### 3.4 Main experiment

#### 3.4.1 Experimental design

This study employed a 2 (conversational partner: human vs. robot) × 2 (strategy: high-efficacy offering specific actions vs. low-efficacy distraction) between-subjects design. Participants were randomly assigned to four conditions: (1) Human × High (n = 4), (2) Human × Low (n = 4), (3) Robot × High (n = 5), and (4) Robot × Low (n = 5). The experiment lasted 6 weeks, with one session held per week. Each session lasted 30 min including questionnaires; the conversation lasted 5–10 min.

#### 3.4.2 Participants

A total of 21 older Japanese adults were recruited. No formal *a priori* power analysis was performed; the sample size was informed by the scale typical of existing longitudinal HRI research (Matheus et al., 2025) and determined primarily by the recruitment and retention constraints associated with the study schedule, which required participants to attend weekly face-to-face, one-to-one conversational sessions for six consecutive weeks. To ensure participant safety, a two-stage screening procedure was implemented: an online questionnaire phase used the Geriatric Depression Scale (GDS-15; Sheikh and Yesavage, 1986), the Kessler Psychological Distress Scale (K6; Kessler et al., 2002), and the AD8 Dementia Screening Interview (AD8; Galvin et al., 2005), completed by a family member or informant, to preliminarily exclude individuals with potential emotional or cognitive risk; on-site, cognitive function was confirmed using Hasegawa’s Dementia Scale–Revised (HDS-R; Kato et al., 1991). These screening measures were used for risk management purposes only and did not constitute clinical diagnoses. Of the 21 recruited participants, one did not meet the screening criteria. The remaining 20 participants were randomly assigned in equal numbers (n = 5 per condition) to the four experimental conditions. Following randomization, two participants withdrew from the study for personal reasons, resulting in a final sample of 18 (Human × High: n = 4; Human × Low: n = 4; Robot × High: n = 5; Robot × Low: n = 5; 8 male, 10 female; age range: 62–90; M = 74.7, SD = 6.4). All were native Japanese speakers recruited through local community activity groups.

Prior to the experiment, participants completed a background questionnaire on demographics and life context. Among the 18 participants, 13 lived with a spouse, three lived alone, two lived with an adult child (one with spouse, one alone). Self-rated health: 15 reported “fairly healthy,” two “very healthy,” one “not very healthy.” All owned smartphones and used them daily; AI or robot conversation experience was limited (12 “none,” four “almost none,” two “occasionally”). Daily conversation frequency: 10 reported conversing with family or friends almost daily, five reported 3–4 days/week, three reported 1–2 days/week. Detailed participant characteristics by condition are presented in Table 3; the full participant-level dataset is provided in Supplementary Table S1.

**TABLE3:** Participant demographics and background characteristics by condition.

Subject ID	Condition	Gender	Age	AI/Robot experience
Human_High1	Human × High	F	74	None
Human_High2	Human × High	M	76	Almost none
Human_High3	Human × High	M	74	None
Human_High4	Human × High	F	78	None
Human_Low1	Human × Low	M	90	Almost none
Human_Low2	Human × Low	F	80	Occasionally
Human_Low3	Human × Low	F	70	None
Human_Low4	Human × Low	M	74	None
Robot_High1	Robot × High	F	67	None
Robot_High2	Robot × High	F	72	Almost none
Robot_High3	Robot × High	F	80	None
Robot_High4	Robot × High	M	62	Almost none
Robot_High5	Robot × High	M	76	None
Robot_Low1	Robot × Low	F	84	None
Robot_Low2	Robot × Low	M	73	None
Robot_Low3	Robot × Low	F	70	None
Robot_Low4	Robot × Low	M	69	Occasionally
Robot_Low5	Robot × Low	F	76	None

#### 3.4.3 Experimental setting and conversational partners

Experimental Setting. The experiment was conducted at a community center in Tokorozawa, Japan. This venue was chosen over a laboratory to reduce anxiety and better approximate natural conversation. Only the participant, experimenter, and (in the human condition) human partner were present; all sessions were audio- and video-recorded. Figure 2 shows the interaction setup: (a) participant with RoBoHoN, (b) participant with human partner.

**FIGURE 2:**
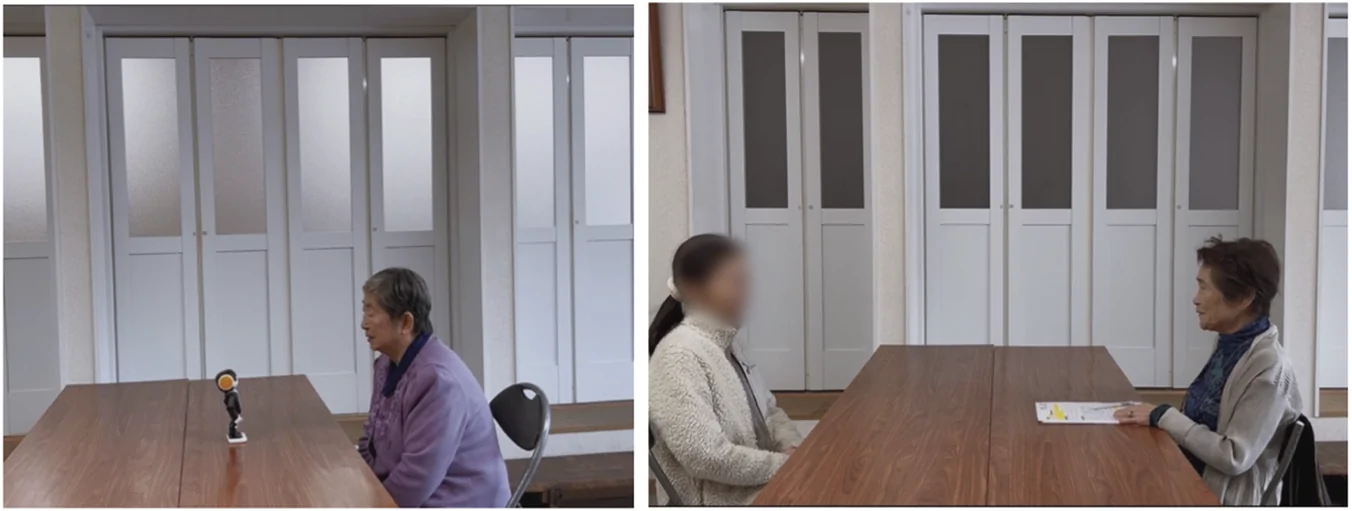
Experimental setting.

Human Partner. In the human condition, two female partners (40s and 50s, recruited via a staffing agency) served as conversational partners, one interacting with seven participants, the other with one. None of the participants had prior acquaintance with the partners before the experiment. Female partners were selected because women’s emotional support tends to be more readily trusted and accepted (Sprecher and Hendrick, 2004). Both were native Japanese speakers trained on dialogue scripts for both encouragement conditions prior to the experiment (see Section 3.4.5). To minimize contact outside the experimental sessions, the human partner entered only when the conversation began and left immediately after; no interaction occurred outside sessions, and participants were prohibited from exchanging contact information.

Robot System. The robot condition used a physical social robot named RoBoHoN (Sharp Corporation; Figure 3). RoBoHoN was selected for three reasons. First, its physical presence enhances perception of it as a human-like entity and strengthens emotional connection, compared with virtual agents that lack visual representation (Li, 2015; Deng et al., 2019). Second, its anthropomorphic design facilitates deeper emotional exchange and may improve recall of conversational content (Beun et al., 2003). Third, its compact, child-like appearance is well-received by older adults in Japan (Figueroa et al., 2023), making it a contextually appropriate choice for this population. RoBoHoN is a small humanoid robot (19.5 cm, 395 g) powered by Android, capable of speech, movement, and LED eye expressions, with a built-in microphone and speaker. None of the participants had prior experience interacting with RoBoHoN. To ensure no outside contact, the robot was present only during the conversation. To focus the comparison on the effects of verbal encouragement, all body movements were disabled; only the eye LED (yellow during conversation) was retained to preserve the robot’s social appearance. Conversations were managed by a custom dialogue system described below.

**FIGURE 3:**
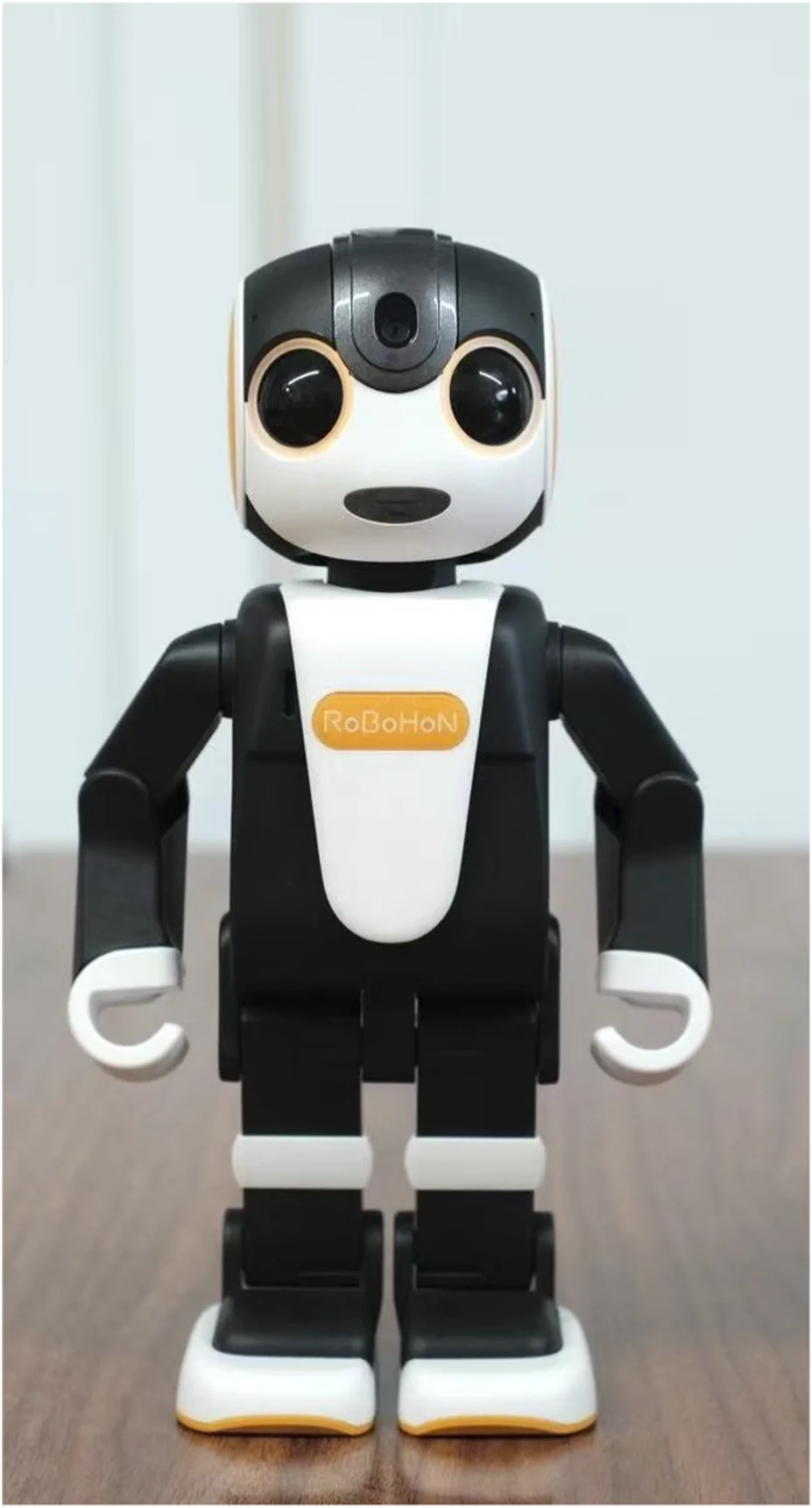
RoBoHoN.

The dialogue system consisted of an Android client on RoBoHoN and a local Python server. Participants’ speech was captured via RoBoHoN’s microphone and recorded in mono at 16 kHz (16-bit PCM). For Voice Activity Detection (VAD), the system performed 3 seconds of ambient noise calibration at each session’s start, collecting 300 audio frames and computing the RMS baseline. Two thresholds were set: high (baseline +400) to trigger recording, and low (baseline +150) to detect silence. Recording began when RMS exceeded the high threshold; when RMS stayed below the low threshold for 300 ms, the utterance was considered complete and uploaded to the server. Upon receiving audio, the server called Google Speech-to-Text API (Japanese, 16 kHz), then invoked OpenAI API (GPT-4o, temperature = 0.7, max_tokens = 200) to generate a response based on the dialogue turn and encouragement condition. The response was synthesized using Google Text-to-Speech API and played back on RoBoHoN. Voice parameters were adjusted (pitch = 1.15, speech rate = 0.9) to approximate the human partner’s voice, minimizing acoustic differences.

At each turn, the prompt supplied to GPT-4o comprised three components: a fixed system prompt, server-managed dialogue context, and a turn-specific dialogue objective. The fixed system prompt, which was identical across all turns, weeks, and conditions, defined the robot’s conversational role, linguistic style, and behavioral constraints. It specified polite Japanese (desu/masu style), a friendly and gentle delivery with natural conversational fillers, and instructions to respond warmly to participants’ worries while avoiding excessive empathy and evaluative expressions. The server managed the dialogue context by tracking the current turn and assigned condition and supplying the preceding dialogue history with each request. The turn-specific objective specified the communicative goal of the turn together with constraints on response structure, length, and wording. This framework allowed each response to build on the participant’s preceding utterances while maintaining the predefined dialogue flow. The prompt architecture and turn-specific objective templates were predefined. Although the dialogue context was updated with each participant response, the system prompt and objective templates were not adaptively modified using any learning algorithm. The overall prompting framework is illustrated in Figure 4. The turn-specific dialogue objectives and encouragement conditions are described in Section 3.4.5, and additional details on prompt construction and dialogue management are provided in the Supplementary Material, “Section 3. Prompt Design for the Robot Dialogue System.”

**FIGURE 4:**
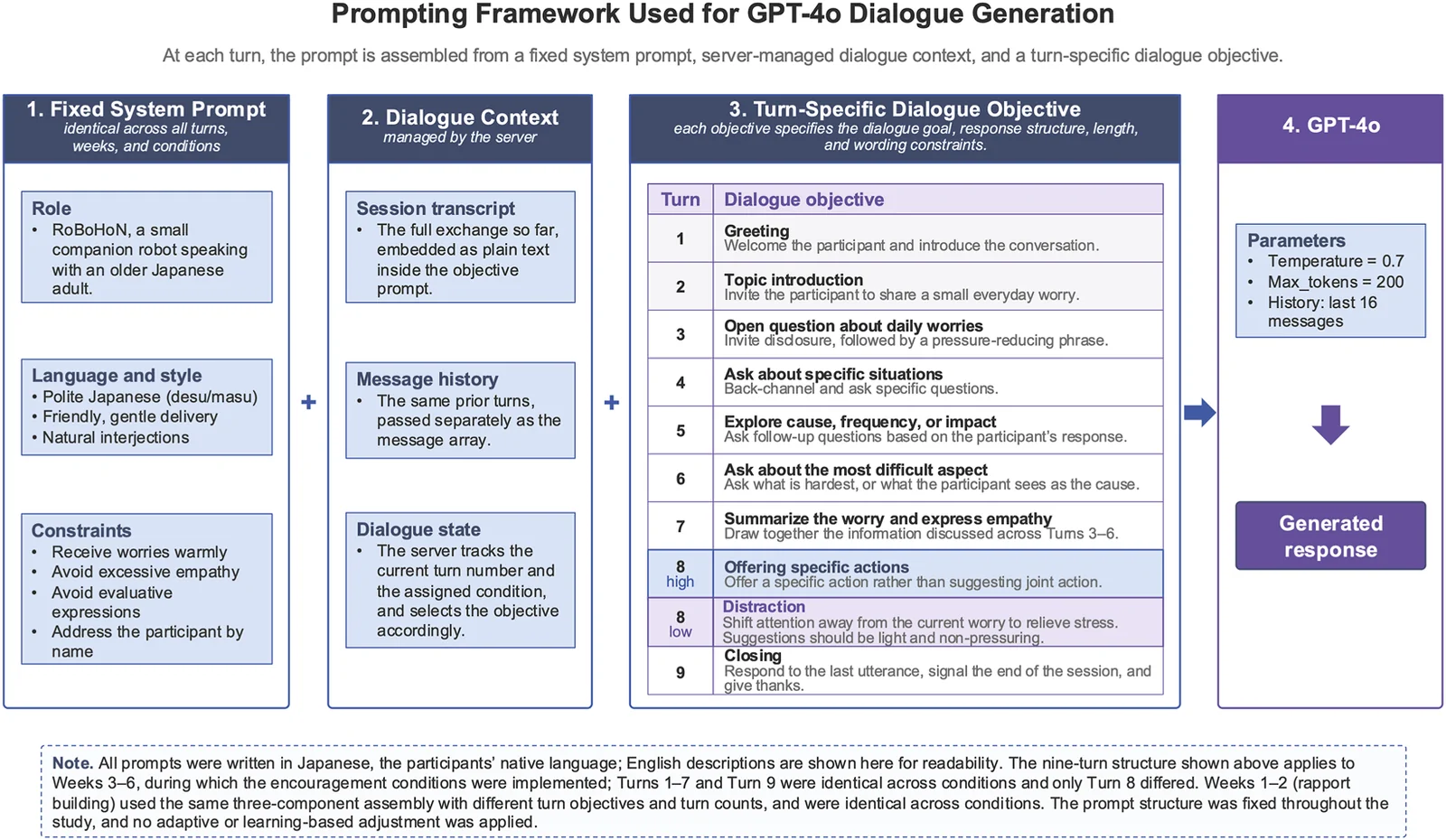
Prompting framework.

#### 3.4.4 Procedure and measures

Conversations were conducted once per week for 6 weeks. Weeks 1–2 served as rapport-building; Weeks 3–6 as encouragement implementation. Rapport-building was included for two reasons.

First, encouragement effectiveness depends on trust and familiarity between the parties involved (Horvath and Greenberg, 1989). Second, the preliminary survey assumed a familiar partner, so the main experiment required a relational foundation for valid strategy comparison. Across all conditions, the human partner and robot were matched on turns, topic structure, and utterance types (see Section 3.4.5). Conversation content was limited to mild everyday worries, excluding clinically significant problems.

Each session proceeded as follows. Upon arrival, the experimenter introduced the conversation topic and provided a keyword list to help participants reflect in advance and facilitate smooth conversation. Participants then engaged in a 5–10 min conversation. Immediately after, they completed a post-session questionnaire.

SWB was assessed at four time points: pre-intervention, after Weeks 2, 4, and 6. AWB was measured using the WHO-5 (Topp et al., 2015), a five-item scale (e.g., “I have felt cheerful and in good spirits”) rated on a six-point scale from 0 (at no time) to 5 (all of the time), with total scores ranging from 0 to 25; higher scores indicate greater AWB. CWB was measured using the SWLS (Diener et al., 1985), a five-item scale (e.g., “In most ways my life is close to my ideal”) rated on a seven-point scale from 1 (strongly disagree) to 7 (strongly agree), with total scores ranging from 5 to 35; higher scores indicate greater CWB.

After each encouragement interaction (Weeks 3–6, four sessions), participants rated perceived encouragement effectiveness on a 0–100 scale (0 = not at all encouraged, 100 = very strongly encouraged). They also provided open-ended responses describing their impressions and reasons.

In addition, participants completed diary entries twice per week (with an interval of three to 4 days), recording daily experiences and emotional states to capture the influence of external life events on SWB.

After the Week 6 session, all participants took part in a post-experiment interview. The interview covered three areas: self-perceived SWB changes and their perceived causes (Q1); multiple-choice attribution of change sources (conversation partner, family/friends, work/hobbies, health, other events, or no change) (Q2); free-response impressions of the conversational experience and partner (Q3).

#### 3.4.5 Dialogue structure and encouragement conditions

The 6-week intervention consisted of a rapport-building phase (Weeks 1–2) and an encouragement implementation phase (Weeks 3–6). The two encouragement conditions (high-efficacy: offering specific actions; low-efficacy: distraction) differed only in Turn 8 of Weeks 3–6; all other turns were identical. Week 1 focused on casual conversation to establish familiarity with the conversational partner through light topics. Week 2 focused on reminiscence-based conversation, which promotes interpersonal closeness and trust through the sharing of personal memories. Reminiscence facilitates relational bonding through affective self-disclosure (Alea and Bluck, 2003), laying a foundation for the worry-sharing sessions in Weeks 3–6. The following describes the turn-by-turn structure for each week.

Week 1 (10 turns). The conversation covered personalized greetings, daily topics, food preferences (with brief agent self-disclosure), hobbies, and a closing with an invitation for the participant to ask questions.

Week 2 (8 turns). The conversation focused on reminiscence, inviting participants to share memories of childhood games, activities, and school life, including exploration of emotional dimensions.

Weeks 3–6 (9 turns). Each week, participants raised one everyday worry. Turns 1–7 established the worry disclosure: greeting, topic introduction, facilitated disclosure, exploration of the worry, follow-up on causes/frequency/impact, confirmation of the most troubling aspect, and empathic summary. Turn 8 was the only turn that differed between conditions: the high-efficacy condition used offering specific actions, and the low-efficacy condition used distraction. Wording varied weekly to avoid repetition, but the functional role of each condition remained consistent across all 4 weeks. All offering specific actions responses were designed to remain within the robot’s actual functional capabilities (e.g., looking up information, setting reminders, or providing a listening presence), rather than implying physical assistance beyond the robot’s capacity. Turn 9 was a closing expression of gratitude with confirmation of the next session.

See Supplementary Material for dialogue scripts with example utterances for each turn.

#### 3.4.6 Data analysis

This study employed a combination of descriptive statistics and content analysis. Given the small sample size (N = 18; n = 4–5 per group), descriptive statistics were used as the primary analytical approach, and no inferential statistical tests were conducted for the main experiment. The analyses focused on characterizing longitudinal patterns within and across conditions using group-level summaries, individual trajectories, and qualitative materials. For AWB (WHO-5), CWB (SWLS), and perceived encouragement effectiveness ratings, group means were calculated at each time point for each condition (Human × High, Human × Low, Robot × High, Robot × Low). These means were used to summarize condition-level patterns, while individual trajectories were examined to characterize within-participant changes and variability across sessions. For AWB and CWB, standardized within-condition changes from pre-intervention to Week 6 were additionally estimated using Cohen’s *d*_z_, calculated as the mean of the individual change scores divided by their standard deviation, together with 95% confidence intervals. For perceived encouragement effectiveness ratings, standardized within-condition changes were estimated in the same way for the change from Week 3, when the encouragement strategies were first implemented, to Week 6. These estimates quantify changes in participants’ evaluations over sustained interaction rather than pre–post intervention effects. Given the small number of participants in each condition, these estimates are reported as descriptive indices of the magnitude and uncertainty of within-condition change rather than as tests of between-condition differences.

For qualitative data, content analysis was applied to post-session open-ended responses, diary entries, and post-experiment interviews. Representative excerpts on encouragement experience, perceived SWB changes, and attribution of changes were identified, extracted, and organized by experimental condition. These qualitative materials were used to explore possible mechanisms underlying the quantitative patterns through participants’ experiences and perceived sources of change, rather than to establish causal relationships. Diary data were used primarily to identify significant external life events and assess whether observed SWB changes may also have been associated with factors outside the conversational intervention.

## 4 Results

The results of the preliminary survey, which determined the two encouragement strategies used in the main experiment, were reported in Section 3.3. This section reports all results obtained during the 6-week main experiment. The results are presented in the following sequence. Changes in affective and cognitive well-being are reported first. The worry topics raised by participants are then described, followed by representative interactions showing how the conversations unfolded in the four experimental conditions. Perceived encouragement effectiveness ratings across Weeks 3–6 are reported next. Finally, qualitative findings from the post-session open-ended responses, post-experiment interviews, and diary entries are presented.

### 4.1 Changes in affective well-being (WHO-5)

Figure 5 and Tables 4, 5 show AWB (WHO-5) changes across four time points (pre-intervention, Week 2, Week 4, and Week 6). Table 5 additionally reports standardized within-condition estimates of change from pre-intervention to Week 6 (Cohen’s *d*_z_ with 95% CIs). Given the small number of participants per condition, these estimates are descriptive and do not indicate between-condition differences or causal effects. The Robot × High group showed the most pronounced increase, with the group mean rising from 15.4 to 19.6 (Δ = +4.2, Cohen’s *d*_z_ = 1.08, 95% CI [−0.09, 2.18]), and most participants showed increases, although the magnitude of change varied. The Robot × Low group showed a modest increase from 18.4 to 19.8 (Δ = +1.4, Cohen’s *d*_z_ = 0.72, 95% CI [−0.31, 1.69]), with considerable individual variation: Robot_Low3 remained stable throughout (17.0→17.0), while Robot_Low1 showed a consistent increase (Δ = +4.0). The Human × High group increased from 16.5 to 18.8 (Δ = +2.3, Cohen’s *d*_z_ = 2.35, 95% CI [0.31, 4.36]); Human_High2 showed a temporary decline at Week 4 followed by recovery, while the other participants showed consistent upward trends. The Human × Low group increased gradually from 16.0 to 17.8 (Δ = +1.8, Cohen’s *d*_z_ = 1.83, 95% CI [0.10, 3.49]).

**FIGURE 5:**
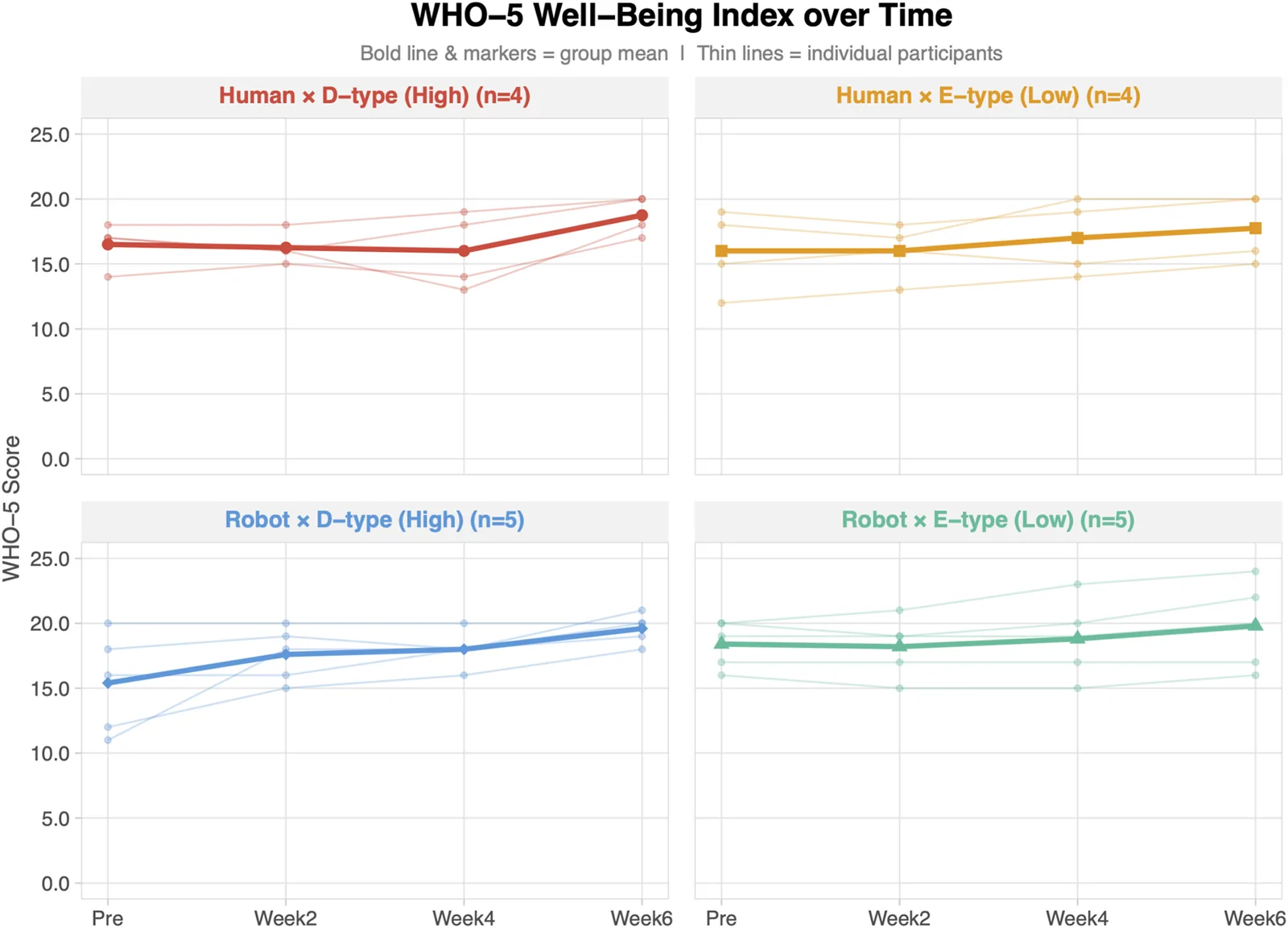
Affective well-being (WHO-5) individual trajectories by condition.

**TABLE4:** Affective well-being (WHO-5) raw scores by participant and condition.

Subject ID	Pre	Week 2	Week 4	Week 6	Δ (Pre→W6)
Human × High					
Human_High1	17	16	18	20	+3
Human_High2	17	16	13	18	+1
Human_High3	18	18	19	20	+2
Human_High4	14	15	14	17	+3
Group mean	16.5	16.3	16.0	18.8	+2.3
Human × Low					
Human_Low1	18	17	20	20	+2
Human_Low2	12	13	14	15	+3
Human_Low3	15	16	15	16	+1
Human_Low4	19	18	19	20	+1
Group mean	16.0	16.0	17.0	17.8	+1.8
Robot × High					
Robot_High1	18	19	18	20	+2
Robot_High2	11	18	18	21	+10
Robot_High3	16	16	18	19	+3
Robot_High4	20	20	20	20	0
Robot_High5	12	15	16	18	+6
Group mean	15.4	17.6	18.0	19.6	+4.2
Robot × Low					
Robot_Low1	20	21	23	24	+4
Robot_Low2	19	19	20	22	+3
Robot_Low3	17	17	17	17	0
Robot_Low4	20	19	19	20	0
Robot_Low5	16	15	15	16	0
Group mean	18.4	18.2	18.8	19.8	+1.4

WHO-5, scores range from 0 to 25; higher scores indicate greater affective well-being. Δ = change from pre-intervention to Week 6. Group means and mean change values are reported to one decimal place. Therefore, minor differences may arise between the displayed Δ and the difference calculated from the displayed pre-intervention and Week 6 means because of rounding.

**TABLE 5:** Within-condition changes and standardized effect-size estimates for affective well-being (WHO-5).

Condition	*n*	*M*(Δ)	*SD*(Δ)	Cohen’s *d*_z_	95% CI
Human × High	4	+2.25	0.96	2.35	[0.31, 4.36]
Human × Low	4	+1.75	0.96	1.83	[0.10, 3.49]
Robot × High	5	+4.20	3.90	1.08	[−0.09, 2.18]
Robot × Low	5	+1.40	1.95	0.72	[−0.31, 1.69]

Δ was calculated as the Week 6 score minus the pre-intervention score. M(Δ) and SD(Δ) represent the mean and standard deviation of the individual change scores, respectively. Cohen’s *d*_z_ was calculated as M(Δ) divided by SD(Δ). The 95% confidence intervals refer to Cohen’s *d*_z_. M(Δ), SD(Δ), Cohen’s *d*_z_, and the corresponding 95% confidence intervals are reported to two decimal places.

All four groups had higher mean WHO-5 scores at Week 6 than at pre-intervention. The mean increase was largest in the Robot × High group (Δ = +4.2) and smallest in the Robot × Low group (Δ = +1.4), whereas the standardized within-condition estimate was largest in the Human × High group (Cohen’s *d*_z_ = 2.35) and smallest in the Robot × Low group (Cohen’s *d*_z_ = 0.72). The difference in Δ between the high- and low-efficacy strategies was 2.8 (4.2–1.4) in the robot condition and 0.5 (2.3–1.8) in the human condition.

### 4.2 Changes in cognitive well-being (SWLS)

Figure 6 and Tables 6, 7 show CWB (SWLS) changes across the same four time points (pre-intervention, Week 2, Week 4, and Week 6). Table 7 additionally reports standardized within-condition estimates of change from pre-intervention to Week 6 (Cohen’s *d*_z_ with 95% CIs). Given the small number of participants per condition, these estimates are descriptive and do not indicate between-condition differences or causal effects. The Human × High group showed the most notable increase, with the group mean rising from 23.0 to 26.3 (Δ = +3.3, Cohen’s *d*_z_ = 0.82, 95% CI [−0.38, 1.94]); Human_High3 showed the largest individual gain (22.0→30.0, Δ = +8.0), while Human_High4 remained stable (23.0→23.0, Δ = 0). The Human × Low group showed a modest increase from 25.0 to 26.3 (Δ = +1.3, Cohen’s *d*_z_ = 0.83, 95% CI [−0.38, 1.96]), with relatively stable individual trajectories. The Robot × High group increased gradually from 23.4 to 25.6 (Δ = +2.2, Cohen’s *d*_z_ = 1.48, 95% CI [0.13, 2.77]), with generally consistent individual trajectories. The Robot × Low group increased from 26.8 to 28.8 (Δ = +2.0, Cohen’s *d*_z_ = 1.26, 95% CI [0.01, 2.45]), with minimal individual variation.

**FIGURE 6:**
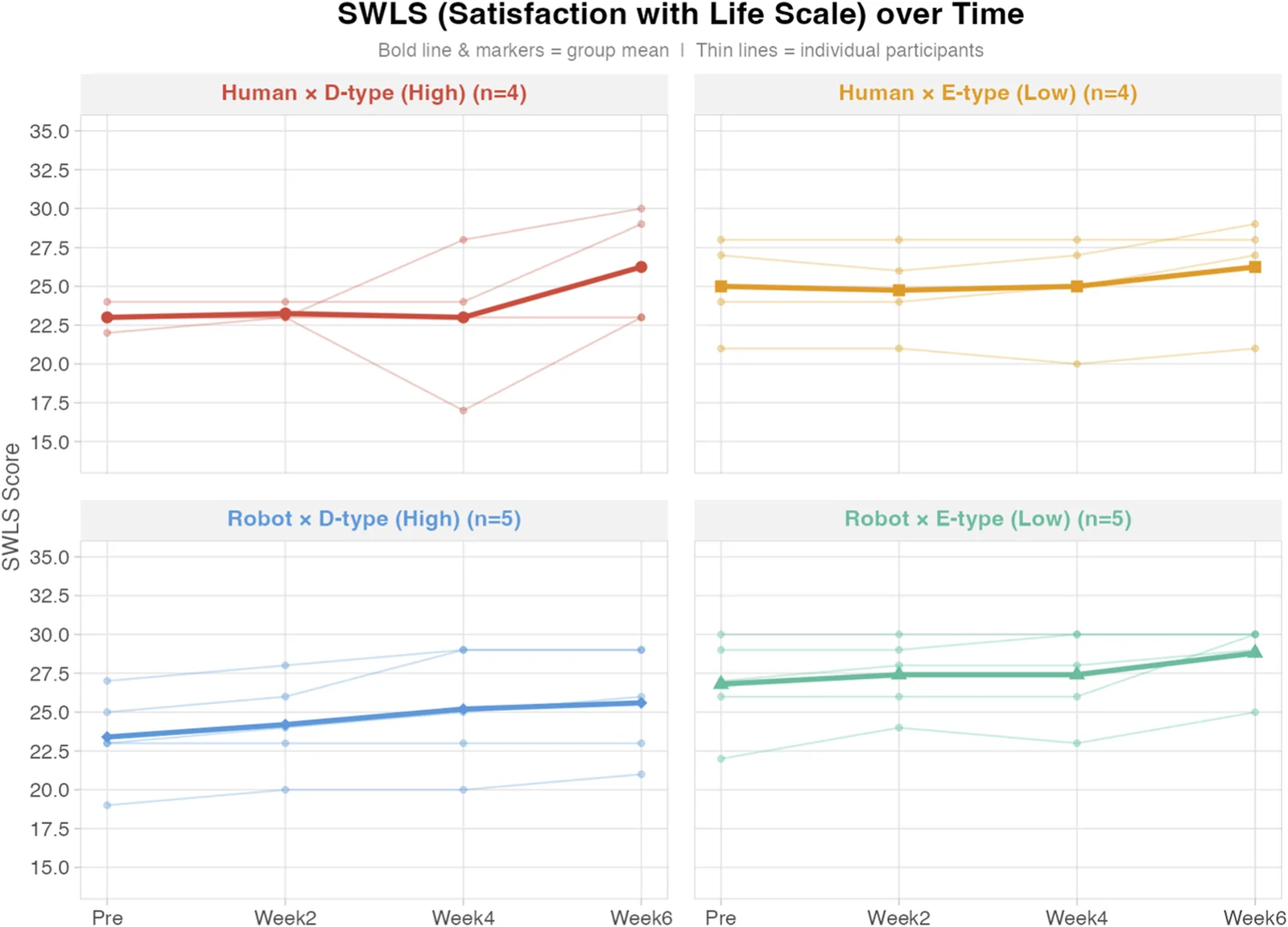
Cognitive well-being (SWLS) individual trajectories by condition.

**TABLE6:** Cognitive well-being (SWLS) raw scores by participant and condition.

Subject ID	Pre	Week 2	Week 4	Week 6	Δ (Pre→W6)
Human × High					
Human_High1	24	24	24	29	+5
Human_High2	23	23	17	23	0
Human_High3	22	23	28	30	+8
Human_High4	23	23	23	23	0
Group mean	23.0	23.3	23.0	26.3	+3.3
Human × Low					
Human_Low1	24	24	25	27	+3
Human_Low2	27	26	27	29	+2
Human_Low3	21	21	20	21	0
Human_Low4	28	28	28	28	0
Group mean	25.0	24.8	25.0	26.3	+1.3
Robot × High					
Robot_High1	23	23	23	23	0
Robot_High2	19	20	20	21	+2
Robot_High3	25	26	29	29	+4
Robot_High4	27	28	29	29	+2
Robot_High5	23	24	25	26	+3
Group mean	23.4	24.2	25.2	25.6	+2.2
Robot × Low					
Robot_Low1	27	28	28	29	+2
Robot_Low2	26	26	26	30	+4
Robot_Low3	30	30	30	30	0
Robot_Low4	29	29	30	30	+1
Robot_Low5	22	24	23	25	+3
Group mean	26.8	27.4	27.4	28.8	+2.0

SWLS, scores range from 5 to 35; higher scores indicate greater life satisfaction. Δ = change from pre-intervention to Week 6. Group means and mean change values are reported to one decimal place. Therefore, minor differences may arise between the displayed Δ and the difference calculated from the displayed pre-intervention and Week 6 means because of rounding.

**TABLE 7:** Within-condition changes and standardized effect-size estimates for cognitive well-being (SWLS).

Condition	*n*	*M*(Δ)	*SD*(Δ)	Cohen’s *d*_z_	95% CI
Human × High	4	+3.25	3.95	0.82	[−0.38, 1.94]
Human × Low	4	+1.25	1.50	0.83	[−0.38, 1.96]
Robot × High	5	+2.20	1.48	1.48	[0.13, 2.77]
Robot × Low	5	+2.00	1.58	1.26	[0.01, 2.45]

Δ was calculated as the Week 6 score minus the pre-intervention score. M(Δ) and SD(Δ) represent the mean and standard deviation of the individual change scores, respectively. Cohen’s *d*_z_ was calculated as M(Δ) divided by SD(Δ). The 95% confidence intervals refer to Cohen’s *d*_z_. M(Δ), SD(Δ), Cohen’s *d*_z_, and the corresponding 95% confidence intervals are reported to two decimal places.

All four groups had higher mean SWLS scores at Week 6 than at pre-intervention. The mean increase was largest in the Human × High group (Δ = +3.3) and smallest in the Human × Low group (Δ = +1.3), whereas the standardized within-condition estimate was largest in the Robot × High group (Cohen’s *d*_z_ = 1.48) and smallest in the Human × High group (Cohen’s *d*_z_ = 0.82). The difference in Δ between the high- and low-efficacy strategies was 2.0 (3.3–1.3) in the human condition and 0.2 (2.2–2.0) in the robot condition.

### 4.3 Worry topics

Across Weeks 3–6, participants reported a total of 72 daily worries (18 participants × 4 weeks). Worry content could be broadly classified into four categories: health and physical concerns (e.g., chronic pain, sleep difficulties, declining fitness), daily life and household burdens (e.g., cleaning, caregiving, domestic friction), interpersonal and social concerns (e.g., conflicts within community groups, tensions involving money), and aging- and loneliness-related concerns (e.g., reduced contact with adult children, anxiety about end-of-life matters). No systematic concentration of any single worry type was observed in any condition group.

### 4.4 Example interactions across the four conditions

Table 8 presents representative nine-turn interactions from each of the four experimental conditions: Human × High, Human × Low, Robot × High, and Robot × Low. In each example, Turns 1–7 elicited and explored an everyday worry, Turn 8 implemented the assigned encouragement strategy, and Turn 9 closed the interaction.

**TABLE 8:** Example interactions across the four experimental conditions.

Turn	Speaker	Utterance
(A) Robot × High		
1	Robot	Hello, [Name]-san. I’m RoBoHoN. I’m glad to speak with you again today. Thank you for joining me
	Participant	Hello. It’s good to speak with you again too
2	Robot	Today, I’d like to hear about a small worry or concern from your daily life. You may talk about anything you feel comfortable sharing. Please let me know when you’re ready
	Participant	I’m ready
3	Robot	Has anything in your daily life been bothering or worrying you recently? Please share only what you feel comfortable discussing
	Participant	I often have to speak in front of fairly large groups, and I worry that I don't speak very well
4	Robot	I see. Are there particular situations or moments when speaking in front of a group feels especially difficult?
	Participant	I usually memorize what I want to say beforehand, but I often get stuck halfway through. I wish I could speak more smoothly
5	Robot	How often does that happen, and how does it affect you when it does?
	Participant	It happens almost every time I speak to a large group. When I see other people speaking so well, I feel discouraged
6	Robot	What is the most difficult part for you?
	Participant	The hardest part is expressing my true feelings honestly without hiding them
7	Robot	So, even after preparing what you want to say, you sometimes get stuck, compare yourself with more fluent speakers, and then find it difficult to express your true feelings. That sounds frustrating
	Participant	Yes, that’s exactly right
8	Robot	I understand. The next time you have to speak in front of a group, please tell me about it. I’ll listen while you practice what you want to say and cheer you on before the presentation
	Participant	I’d be glad to have your encouragement, RoBoHoN. I’ll talk with you again next time
9	Robot	Of course. We’re almost out of time, [Name]-san. Thank you for sharing your concern with me today. I look forward to speaking with you again
	Participant	Okay, thanks
(B) Robot × Low		
1	Robot	Hello, [Name]-san. I’m RoBoHoN. I’m glad to speak with you again today. Thank you for joining me
	Participant	It’s good to speak with you again too
2	Robot	Today, I’d like to hear about a small worry or concern from your daily life. You may talk about anything you feel comfortable sharing. Please let me know when you’re ready
	Participant	Okay, I’m ready
3	Robot	Has anything in your daily life been bothering or worrying you recently? Please share only what you feel comfortable discussing
	Participant	There are about 15 members in my ocarina group, and most of them are older adults. Many are around 75, and some are over 80. One member sometimes becomes emotional and says harsh things. I’m worried about how to deal with that person
4	Robot	I see. In what situations does this person tend to speak harshly to others?
	Participant	For example, everyone occasionally plays a wrong note, and most members are understanding. However, this person loudly criticizes others when they make mistakes, even though they also make mistakes themselves. I find that difficult
5	Robot	Why do you think this person reacts that way?
	Participant	I think it may simply be part of that person’s personality
6	Robot	What is the most difficult part of dealing with this situation?
	Participant	I’m not used to being criticized in such an emotional manner, so I become flustered. That is what I find most difficult. I also wonder how I or the group should respond
7	Robot	So, when this person criticizes others harshly in the ocarina group, you feel flustered because you are not used to being spoken to that way. That sounds difficult
	Participant	Yes, exactly
8	Robot	I see. How about having some warm soup? The warmth may help you relax and feel a little lighter
	Participant	Oh, that sounds nice. Thank you
9	Robot	I’m glad that sounds good. Thank you for sharing your concern with me today. I look forward to speaking with you again
	Participant	Okay
(C) Human × High		
1	Human partner	Hello, [Name]-san. It’s good to speak with you again today. Thank you for joining me
	Participant	It’s good to speak with you again too
2	Human partner	Today, I’d like to hear about a small worry or concern from your daily life. Please let me know when you’re ready
	Participant	Okay, I’m ready
3	Human partner	Could you tell me about something that has been bothering or concerning you recently? Please share only what you feel comfortable discussing
	Participant	I’m currently involved in three volunteer groups and two hobby clubs, and I feel a little overwhelmed. Yesterday, three activities overlapped, so I could attend only two. Each activity is important to me, but I’m getting older and feel very tired. I’m thinking of reducing some of my commitments next year
4	Human partner	When do the schedules tend to overlap, and which activities are hardest for you to adjust?
	Participant	I’ve been involved in one volunteer group for about five or 6 years and another for about 2 years. I also belong to two hobby clubs, so the schedules often overlap
5	Human partner	How often do you have activities scheduled on consecutive days, and how does that affect you?
	Participant	This week, I have something scheduled every day from Monday to Friday. I wish I had a little more time to relax. I think having time to do nothing is important, because I always feel as though I’m being chased by my schedule
6	Human partner	What feels most difficult: the physical fatigue, the overlapping schedules, or having almost no time to rest?
	Participant	I think having almost no time to rest is the hardest part. I enjoy the activities, but I would like a little more free time
7	Human partner	So, the activities are important and enjoyable to you, but the overlapping commitments and lack of rest leave you tired. It sounds difficult to find a balance without giving up things you value
	Participant	Yes, exactly
8	Human partner	I will look up some simple scheduling methods and ways to organize your commitments and share them with you. I hope that will make it easier to see which commitments you could reduce or rearrange
	Participant	Thank you
9	Human partner	I’m glad to help. Thank you very much for sharing your concern with me today
	Participant	It may not be a major worry, but thank you
(D) Human × Low		
1	Human partner	Hello, [Name]-san. It’s good to speak with you again today. Thank you for joining me
	Participant	It’s good to speak with you again too
2	Human partner	Today, I’d like to hear about a small worry or concern from your daily life. Please let me know when you’re ready
	Participant	Okay, I’m ready
3	Human partner	Could you tell me about something that has been bothering or concerning you recently? Please share only what you feel comfortable discussing
	Participant	I’d like to talk about pets. I used to have two cats, but they went missing a few years ago around the time of the pandemic. The family upstairs got a toy poodle 2 months ago. It sometimes comes over to play, which I enjoy. Because they are often at work and the puppy is still young, they ask us to feed it. We are not always at home, so coordinating the feeding times can be difficult
4	Human partner	I see. Are the feeding times fixed, and in what situations is it difficult to keep to them?
	Participant	The feeding times are usually fixed in the morning and evening, and sometimes around noon. Since we are not always at home, the timing sometimes shifts. They say that is fine, but I still worry about whether it is really all right
5	Human partner	How often do you need to feed the puppy, and how much does that affect your schedule?
	Participant	We feed it almost every day. Once it gets a little older, two meals a day should be enough. It can be inconvenient at times, but I also find it enjoyable
6	Human partner	What is the most difficult part for you: having to be home around noon, adjusting your outings, or worrying about the puppy after you leave?
	Participant	The main difficulty is making sure I am home around noon. I sometimes have to come home specifically to feed it. Afterward, I play with it for a while and then put it back in its crate, but I feel reluctant to leave and wonder whether it is sleeping comfortably
7	Human partner	So, you enjoy helping care for the puppy, but needing to return around noon sometimes disrupts your plans, and you also worry about leaving it alone afterward. That sounds difficult to manage
	Participant	Yes, that’s right
8	Human partner	I see. How about having a cup of warm herbal tea to take your mind off things? Chamomile tea may help you relax
	Participant	Yes, that sounds nice
9	Human partner	I’m glad that sounds good. Thank you very much for sharing your concern with me today
	Participant	Thank you

The original interactions were conducted in Japanese and translated into English while preserving the original meaning as closely as possible.

### 4.5 Perceived encouragement effectiveness

Figure 7 and Tables 9,10 show perceived encouragement effectiveness ratings (0–100) across Weeks 3–6. Table 10 additionally reports standardized within-condition estimates of change from Week 3 to Week 6 (Cohen’s *d*_z_ with 95% CIs). Given the small number of participants per condition, these estimates are descriptive and do not indicate between-condition differences or causal effects.

**FIGURE 7:**
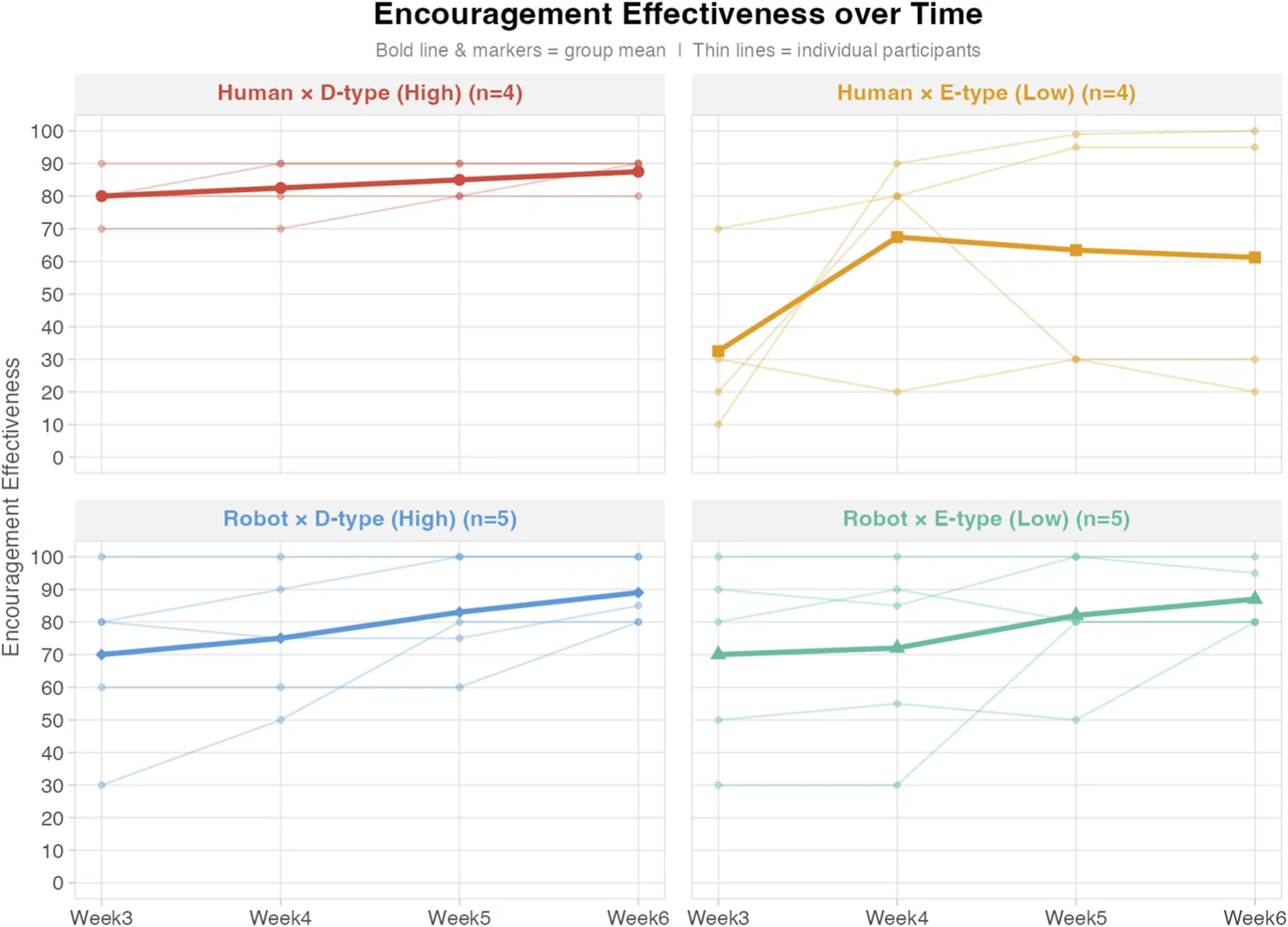
Perceived encouragement effectiveness by strategy type and condition across weeks 3–6.

**TABLE9:** Perceived encouragement effectiveness ratings by participant and condition (Weeks 3–6).

Subject ID	Week 3	Week 4	Week 5	Week 6	Δ (W3→W6)
Human × High					
Human_High1	90	90	90	90	0
Human_High2	70	70	80	90	+20
Human_High3	80	90	90	90	+10
Human_High4	80	80	80	80	0
Group mean	80.0	82.5	85.0	87.5	+7.5
Human × Low					
Human_Low1	30	20	30	30	0
Human_Low2	10	90	99	100	+90
Human_Low3	70	80	95	95	+25
Human_Low4	20	80	30	20	0
Group mean	32.5	67.5	63.5	61.3	+28.8
Robot × High					
Robot_High1	30	50	80	80	+50
Robot_High2	80	90	100	100	+20
Robot_High3	100	100	100	100	0
Robot_High4	80	75	75	85	+5
Robot_High5	60	60	60	80	+20
Group mean	70.0	75.0	83.0	89.0	+19.0
Robot × Low					
Robot_Low1	30	30	80	80	+50
Robot_Low2	100	100	100	100	0
Robot_Low3	50	55	50	80	+30
Robot_Low4	80	90	80	80	0
Robot_Low5	90	85	100	95	+5
Group mean	70.0	72.0	82.0	87.0	+17.0

Ratings were made on a 0–100 scale (0 = not at all encouraged, 100 = very strongly encouraged) immediately after each encouragement interaction. Δ = change from Week 3 to Week 6. Group means and mean change values are reported to one decimal place. Therefore, minor differences may arise between the displayed Δ and the difference calculated from the displayed Week 3 and Week 6 means because of rounding.

**TABLE 10:** Within-condition changes and standardized effect-size estimates for perceived encouragement effectiveness (Weeks 3–6).

Condition	*n*	*M*(Δ)	*SD*(Δ)	Cohen’s *d*_z_	95% CI
Human × High	4	+7.50	9.57	0.78	[−0.41, 1.89]
Human × Low	4	+28.75	42.50	0.68	[−0.47, 1.74]
Robot × High	5	+19.00	19.49	0.97	[−0.15, 2.03]
Robot × Low	5	+17.00	22.25	0.76	[−0.28, 1.75]

Δ was calculated as the Week 6 rating minus the Week 3 rating. M(Δ) and SD(Δ) represent the mean and standard deviation of the individual change scores, respectively. Cohen’s *d*_z_ was calculated as M(Δ) divided by SD(Δ). The 95% confidence intervals refer to Cohen’s *d*_z_. M(Δ), SD(Δ), Cohen’s *d*_z_, and the corresponding 95% confidence intervals are reported to two decimal places.

The Human × High group maintained consistently high ratings, with the group mean rising from 80.0 to 87.5 (Δ = +7.5, Cohen’s *d*_z_ = 0.78, 95% CI [−0.41, 1.89]) and small individual variation.

The Human × Low group showed the most pronounced individual variability, with the group mean increasing from 32.5 to 61.3 (Δ = +28.8, Cohen’s *d*_z_ = 0.68, 95% CI [−0.47, 1.74]); however, this mean masks substantial individual differences, with some participants showing sharp increases and others returning to low ratings by Week 6. The Robot × High group showed a steady increase from 70.0 to 89.0 (Δ = +19.0, Cohen’s *d*_z_ = 0.97, 95% CI [−0.15, 2.03]), with several participants starting relatively low and increasing progressively. The Robot × Low group showed a similar increase from 70.0 to 87.0 (Δ = +17.0, Cohen’s *d*_z_ = 0.76, 95% CI [−0.28, 1.75]), with consistently high ratings maintained by multiple participants throughout.

All four groups had higher mean perceived encouragement effectiveness ratings at Week 6 than at Week 3, although fluctuations were observed between sessions. The mean increase was largest in the Human × Low group (Δ = +28.8) and smallest in the Human × High group (Δ = +7.5), whereas the standardized within-condition estimate was largest in the Robot × High group (Cohen’s *d*_z_ = 0.97) and smallest in the Human × Low group (Cohen’s *d*_z_ = 0.68). At Week 6, the difference between the high- and low-efficacy strategies was 26.2 (87.5–61.3) in the human condition and 2.0 (89.0–87.0) in the robot condition.

### 4.6 Qualitative findings

First, content analysis of the post-session open-ended responses yielded four findings.

Finding 1. Worries disclosed during the sessions were rarely discussed in daily life. Many participants reported that the worries they shared during the sessions had rarely or never been disclosed to others in daily life. Reasons for non-disclosure were consistent across conditions: not wanting to burden others (Human_Low2: “it becomes a complaint, and the listener suffers”; Robot_Low3: “with a human listener, I cannot say things that might burden them”), perceiving concerns as too trivial (Human_High4: “because it is such a trivial everyday matter, I do not talk about it with friends”), lacking suitable confidants, and believing that others would not understand (Human_High1: “even if I told someone outside, they would not understand”). This pattern was observed regardless of partner or strategy type. Human_High3 noted the topic had not been shared with friends; Robot_High3 reported sharing the content for the first time; Robot_Low1 stated that private matters were not shared; and Robot_Low4 noted that small worries were almost never discussed with friends. Reports of feeling at ease during disclosure were more common among participants in the robot conditions. Human_Low4 stated: “just having someone listen and respond is enough to make me happy”; Robot_Low4 noted: “it is good to have someone I can talk to about my complaints.” Positive comments about being listened to were reported across all four conditions.

Finding 2. Participants’ comments on the high- and low-efficacy strategies differed across partner conditions. In the human condition, participants receiving the high-efficacy strategy (offering specific actions) referred both to the concrete actions themselves and to a sense of being valued. Human_High1 noted that specific actions were more reassuring than simple comfort; Human_High3 reported that a promise to look into possible solutions provided motivation; and Human_High4 repeatedly expressed appreciation for concrete actions and reported feeling very pleased upon receiving them. In the robot condition, participants’ evaluations of the high-efficacy strategy varied according to the content of the worry. Robot_High1 reported a perceived capability mismatch when the worry involved physical issues, stating: “the robot is not actually going to cook for me” and “I already know this, so it is unnecessary.” In contrast, Robot_High2 and Robot_High5 responded positively when the worry involved psychological or interpersonal issues, expressing a desire to think through solutions together and feeling that the robot genuinely understood their situations.

For the low-efficacy strategy (distraction), Robot_Low2 reported that a suggestion to take a bath helped him calm down and reconsider the day’s events; Human_Low3 found that light suggestions were easier to act upon. Participants’ comments also referred to the match between the suggested content and their personal habits (Robot_Low1 rated a movie suggestion at 30 points, stating that a music suggestion would have received 100 points), as well as to perceived repetition (Robot_Low5 rated a suggestion lower because it felt similar to the previous week’s, despite being objectively different).

Finding 3. Participants reported changes in their thinking after verbalizing their worries. Multiple participants reported that verbalizing and sharing their worries led to a renewed understanding of their concerns. Human_High3 stated that “it was a good opportunity to reflect on myself, and I realized there was another way of looking at things,” and reported that after the session, “my worries were organized and I could see a way forward.” Human_High2 expressed a desire to discuss life goals and personal reflection more deeply. Robot_High4 described the process of verbalization: “in order to express my vague feelings, I first had to put them into words, and then RoBoHoN understood and showed empathy. This entire process functioned as a meaningful form of communication.”

Finding 4. Multiple robot-condition participants responded positively to RoBoHoN’s visual feedback features, particularly the illuminated eyes. Robot_High1 explicitly stated: “it feels like it is truly listening to me.” Robot_High2, Robot_High4, Robot_Low1, Robot_Low2, and Robot_Low3 expressed similar impressions, reporting that the illuminated eyes made them feel that the robot was attentively listening.

Next, content analysis of the post-experiment interviews and diaries yielded four findings. Finding 5 is based on Q1 (self-perceived SWB changes and causes) and Q2 (multiple-choice attribution); Findings 6 and 7 are based on Q3 (free-response impressions); Finding 8 is based on diary data.

Finding 5. Most participants identified the conversational sessions as a source of perceived SWB change, although some participants’ interview responses and questionnaire score trajectories were not fully aligned. In Q2, 15 of 18 participants (83.3%) identified conversations as a source of SWB change, including all four in Human × High and all five in Robot × High; three in each of Human × Low and Robot × Low also selected this option. The remaining three who did not select this option were Human_Low1, Robot_Low1, and Robot_Low5. Beyond the sessions, community activities and hobbies were the second most frequent attribution source (n = 8), followed by family/friend events (n = 3), health changes (n = 3), and other daily events (n = 3).

Human_Low1 reported no perceived change in Q1 and did not select the conversational sessions as a source of change in Q2, although both the participant’s WHO-5 and SWLS scores increased from pre-intervention to Week 6. Robot_Low1 attributed perceived change to the conversational sessions in Q1 but did not select the conversational sessions as a source of change in Q2; both the participant’s WHO-5 and SWLS scores increased from pre-intervention to Week 6.

Finding 6. Some participants reflected on situations suited to human and robot partners. In Q3, some participants spontaneously reflected on what each partner type might be better suited for, based on their own experience with that partner. Human_Low1 noted: “for emotionally sensitive matters, a human is better; for more objective things, a robot might be more appropriate.” Robot_High4 noted that a robot should distinguish between when advice is needed and when the person simply wants to be heard. These two participants were in different conditions and unaware of each other’s responses.

Finding 7. Participants reported different partner-related impressions in Q3. Responses from participants in the human conditions included expressions related to feeling understood, relational depth, and self-reflection. Human_Low1 noted emotionally sensitive matters were better discussed with a human, and Human_High2 expressed a desire to explore life goals and personal reflection more deeply. Responses from participants in the robot conditions included expressions related to ease and closeness. Robot_Low4 described the robot as a “companion,” Robot_Low5 reported “familiarity, closeness, and trust,” and Robot_High3 noted the robot “initially seemed childlike, but its way of thinking felt mature.”

Finding 8. Diary entries recorded external life events occurring during the study period. Diary data indicated that all participants experienced a range of everyday events during the study period, including community activities, travel, family gatherings, and seasonal events. Some participants experienced events with a more substantial emotional impact: Human_High2 recorded the death of an acquaintance and noted having “thought about many things”; Robot_Low5 reported that “a friend’s husband passed away, which was a shock.” Both participants’ SWB trajectories showed a temporary fluctuation at the corresponding time points. The diary entries documented the external events that coincided with these individual score fluctuations.

## 5 Discussion

This study investigated the effects of high-efficacy (offering specific actions) and low-efficacy (distraction) encouragement on older Japanese adults’ SWB over 6 weeks, comparing human-delivered and robot-delivered encouragement (RoBoHoN). A mixed-methods design combined descriptive statistics with content analysis of post-session responses, diaries, and interviews to address RQ1 (SWB changes) and RQ2 (perceived encouragement effectiveness over time). The following sections interpret the key findings and discuss their implications for each research question, followed by limitations, future directions, and the conclusion.

### 5.1 Longitudinal changes in SWB by strategy and partner (RQ1)

#### 5.1.1 Interpretation of key findings

In response to RQ1, two main findings were identified. First, mean AWB and CWB scores at Week 6 were higher than at the pre-intervention assessment in all four conditions. Second, the difference between the high- and low-efficacy strategies showed a crossover-like pattern. The difference was more evident for AWB in the robot condition and for CWB in the human condition. Given the small number of participants per condition, these patterns are based on descriptive comparisons and should be interpreted accordingly. AWB and CWB reflect the affective and cognitive components of SWB, respectively (Diener et al., 1985; Topp et al., 2015). The findings therefore suggest that the association between encouragement and SWB may depend on both the conversational partner and the component of SWB examined.

For AWB, the high–low difference was larger in the robot condition. One possible explanation is that people may have fewer concerns about social evaluation when interacting with an artificial agent than with a human (Lucas et al., 2014; Holthöwer and van Doorn, 2023). In the present study, participants in both robot conditions similarly reported feeling at ease when disclosing their worries (Finding 1). This shared low-pressure setting may have contributed to the overall positive affective experience of robot interaction, operating more directly on the hedonic dimension reflected in AWB, that is, the affective component of SWB captured by the WHO-5 (Topp et al., 2015). However, because this setting was common to both robot conditions, it cannot by itself explain the difference between the two strategies. Meanwhile, Robot × High showed a larger mean increase than Robot × Low (Δ = +4.2 vs. +1.4), the two groups had nearly identical scores at Week 6 (19.6 vs. 19.8), and Robot × High started from a lower baseline (15.4 vs. 18.4). Therefore, the larger increase in Robot × High may be partly attributable to the lower baseline, rather than the effect of the high-efficacy strategy alone. Nonetheless, the qualitative findings suggest that the strategy itself may also have contributed. Participants in Robot × High reported that offering specific actions helped them consider possible solutions or made them feel that the robot understood their situations (Finding 2). By contrast, distraction redirects attention away from the worry rather than responding directly to it. Therefore, it may be less likely to create a clear sense that the worry itself has been understood and responded to. This difference may partly explain the smaller AWB increase in Robot × Low.

Regarding CWB, the high–low difference was larger in the human condition. According to the transactional model of stress and coping, individuals’ judgments about available coping resources and the manageability of a situation influence how they understand and respond to stressful circumstances (Lazarus and Folkman, 1984). Offering specific actions may make coping options more explicit, thereby changing participants’ perceptions of how manageable their worries are and encouraging them to reconsider their worries in terms of possible ways of addressing them. In the human condition, this mechanism seems to be particularly pronounced. Offering specific actions from a human partner not only provides coping information but may also carry relational meaning, signaling that the other person is willing to engage with the worry. The resulting sense of being valued and socially connected is likely to further influence how participants appraise their situations (Baumeister and Leary, 1995). Participants in the Human × High condition similarly reported that this encouragement reassured them, motivated them, and helped them see a possible way forward (Finding 2). However, the same interpretation may not apply fully when this strategy is delivered by a robot. Some participants considered whether the robot could actually perform the actions it offered (Finding 2). Thus, compared with the same encouragement from a human partner, robot-delivered action offers appear to be less likely to be construed as credible coping resources, which may have weakened their association with participants’ appraisals of manageability. This could partly explain why offering specific actions was less strongly associated with CWB in the robot condition, and consequently why the strategy difference was smaller in that condition. In contrast, distraction neither directly suggests how the worry might be addressed nor conveys the same willingness to engage with it. This may further explain why the two strategies were more clearly differentiated for CWB in the human condition.

In addition, the positive mean changes across all four conditions suggest that changes in SWB may have been related not only to the specific encouragement strategy but also to the supportive conversational process. Prior research has linked emotional self-disclosure to positive psychological and emotional experiences, and high-quality listening to perceived responsiveness, including feeling understood, validated, and cared for (Ho et al., 2018; Itzchakov and Reis, 2023). Most participants rarely discussed the worries raised during the sessions with others, and some reported that verbalizing their worries helped them organize or reconsider their concerns (Findings 1 and 3). For older adults, changes in social roles and networks may reduce everyday opportunities to have their concerns heard and responded to (Carstensen et al., 1999). This may be particularly relevant for the Japanese older adults in this study, as some participants avoided discussing their worries precisely because they did not want to burden others (Finding 1), consistent with cultural norms emphasizing consideration for others (Markus and Kitayama, 1991). Therefore, regular opportunities to express worries and receive listening and responses may have had supportive value in themselves. This may partly explain the positive mean changes observed in the two low-efficacy conditions.

These interpretations should be treated with caution. Diary data indicated that some fluctuations in SWB coincided with external life events (Finding 8), and the absence of a no-intervention control means that the observed changes cannot be attributed entirely to the experimental sessions. With these caveats, the overall pattern suggests that encouragement may relate to SWB in different ways depending on the partner: robot interaction appeared more distinctly associated with AWB, human interaction with CWB, and the conversational process of disclosure and being listened to may have contributed to positive changes across all conditions.

#### 5.1.2 Implications

The findings related to RQ1 have several theoretical and practical implications.

First, this study extends human-robot support research by focusing on encouragement as a specific form of supportive communication, rather than on general counseling, companionship, or open-ended emotional support. Although prior studies have compared human support with chatbot- or agent-based support, most have relied on virtual agents rather than physically embodied robots and have typically lacked a human partner as a direct comparison condition (Li et al., 2026; Matheus et al., 2025). By comparing human-delivered and robot-delivered encouragement in matched strategy conditions, this study provides preliminary evidence that whether encouragement comes from a human or a robot may be associated differently with older adults’ SWB.

Second, the findings suggest that encouragement strategies may relate to SWB dimensions differently, and that interpersonal strategy classifications may not directly apply to HRI. Existing HRI research has largely used composite outcomes or not distinguished AWB from CWB (Yen et al., 2024; Li et al., 2026). The crossover-like pattern suggests the same strategy may show different associations by partner type: offering specific actions was more strongly associated with CWB in the human condition, but with AWB in the robot condition. This suggests that interpersonal support theories may not transfer unchanged to HRI and warrant re-examination.

Third, this partner-dependent pattern is consistent with the Differential Encouragement Framework (Xiang et al., 2025), which proposes that encouragement from humans and agents may be processed through different psychological pathways. The findings further suggest that these pathways may be associated with different SWB components: robot-delivered encouragement may be more closely associated with affective experiences, whereas human-delivered action-oriented encouragement may be more closely associated with cognitive appraisal. This complementary pattern suggests that robot-delivered encouragement may be better understood as a complement to, rather than a replacement for, human relationships.

In addition, the findings suggest that sustained supportive interaction may operate at both the utterance and conversational levels. The positive mean changes across all conditions, together with participants’ accounts of disclosing worries and being listened to, suggest two partially distinct pathways of support: a content-based pathway associated with the specific strategy, and a relational pathway associated with being heard. The latter may be particularly relevant in cultures where concerns about burdening others constrain self-disclosure (Markus and Kitayama, 1991). More broadly, as noted in Section 2.3, the longer-term contribution of social robots may need to be considered not only through sustained changes in standardized outcome measures but also through the interactional and relational roles that robots come to occupy in users’ everyday lives.

Practically, these findings suggest several design considerations for social robots intended to support older adults’ well-being. Such systems should select appropriate encouragement strategies and maintain a conversational process that facilitates disclosure, listening, and responsive feedback across repeated interactions. Human and robot partners should not be assumed to serve identical functions. Robots may provide low-threshold opportunities for everyday support, while human support remains important for concerns beyond robot capabilities. Systems could incorporate a routing mechanism that directs everyday worries to the robot and guides deeper issues to human resources such as family members or professional services. Such routing may also mitigate the risk of overdependence, which may be particularly consequential for older adults whose social networks tend to shrink with age (Carstensen et al., 1999).

### 5.2 Perceived encouragement effectiveness over time and across partners (RQ2)

#### 5.2.1 Interpretation of key findings

In response to RQ2, two main findings were identified. First, Week 6 mean ratings of perceived encouragement effectiveness were at least as high as Week 3 in all conditions, despite fluctuations between sessions. Second, the relative evaluation of the two strategies differed by partner: the strategy distinction observed in the Phase 1 single-session evaluation remained evident in the human condition of Phase 2 (Δ gap = 26.2), whereas in the robot condition the two strategies converged to similarly high ratings (Δ gap = 2.0). Because of the small number of participants per condition, no statistical tests of between-condition differences were conducted, and these patterns should be interpreted as preliminary descriptive trends. The findings therefore suggest that perceived effectiveness may depend not only on the strategy itself but also on the conversational partner.

In the human condition, offering specific actions remained clearly more highly rated than distraction throughout the four sessions. Participants in Human × High responded positively, reporting that they felt reassured, motivated, or better able to see a way forward (Finding 2). As discussed in Section 5.1.1, action-oriented encouragement from a human partner may convey not only the offered actions themselves but also the partner’s willingness to engage with the concern. By contrast, evaluations of distraction varied more across participants. Some found the suggestions lighthearted and enjoyable, while others rated them lower because the suggestions did not match their personal interests or daily habits (Finding 2). Thus, even within the same strategy, perceived effectiveness may depend on the fit between specific content and individual preferences.

Ratings for offering specific actions also showed different trajectories by partner. In the human condition, ratings were high from Week 3 onward (80.0), suggesting that action-oriented offers from a human partner carried credibility from the outset. In the robot condition, ratings started lower (70.0) but rose steadily to 89.0 by Week 6, suggesting that receptiveness to robot-delivered action-oriented encouragement may have developed gradually over sustained interaction.

In the robot condition, evaluations appeared to depend on several forms of fit. For offering specific actions, participants considered whether the offered actions were consistent with their understanding of the robot’s capabilities. Some questioned the plausibility of action-oriented responses to physical worries (e.g., “the robot is not actually going to cook for me”), and may have attributed such statements to system output rather than genuine intent (Nass and Moon, 2000), whereas similar responses to psychological or interpersonal concerns were received more positively (Finding 2). This pattern is consistent with the Phase 1 finding that offering specific actions showed no clear advantage for physical worries. Distraction ratings were also influenced by personal preferences and perceived repetition: even when suggestions were objectively different across sessions, they could be evaluated less positively if participants perceived them as functionally similar to previous suggestions. Robot-delivered encouragement therefore appears to be evaluated not only by strategy category, but also by the fit across factors such as worry type, specific content, user preference, and perceived robot capability.

The convergence of the two strategies in the robot condition further suggests that evaluations may reflect both encouragement content and the broader interactional experience. Content-oriented judgments concern whether a response is relevant, feasible, and suited to the individual, whereas relationship-oriented judgments concern the overall experience of the interaction partner across sustained encounters. Some participants reported familiarity, closeness, and trust toward the robot and described it as companion-like (Finding 7), while RoBoHoN’s illuminated-eye feedback was interpreted by some as a sign of attentive listening (Finding 4). As interaction continued, evaluations of individual encouragement utterances may therefore have become increasingly embedded in participants’ overall impressions of the robot and the interaction. This does not imply that content ceased to matter, but rather that relational experience may have operated alongside content in shaping evaluations. This interpretation is conceptually related to parasocial relationship research (Hoffner and Bond, 2022), although the present study did not directly assess whether such relationships had formed.

Participants also expressed expectations about the appropriate roles of human and robot support providers. Some distinguished between concerns better discussed with a human and those suitable for a robot, and noted that a robot should recognize whether the user wants advice or mainly wishes to be heard (Finding 6). Perceived effectiveness may therefore depend not only on whether a response is helpful, but also on whether it is seen as appropriate for that partner in that situation.

Finally, the generally positive and in some cases rising ratings over the four sessions do not rule out novelty effects. Participants were unfamiliar with RoBoHoN, and the encouragement phase lasted only 4 weeks. The weekly variation in worry topics and response content may have helped sustain engagement. Longer-term studies are therefore needed to determine whether the observed evaluation patterns would persist as novelty diminishes.

Overall, the findings suggest that perceived encouragement effectiveness in sustained interaction may be shaped not only by strategy type, but also by multiple forms of fit between the support content, the user, and the partner, as well as by the user’s evolving understanding of the interaction partner. Because these processes appeared to differ between the human and robot conditions, perceived effectiveness may be better understood as context-dependent, varying with the support provider and the course of interaction, rather than as a fixed property of the encouragement strategy.

#### 5.2.2 Implications

The findings related to RQ2 have several theoretical and practical implications.

First, the findings suggest that perceived encouragement effectiveness is not a stable property of a strategy, but may develop through sustained interaction with a particular support provider. The divergent evaluation patterns observed in the human and robot conditions suggest that how users evaluate encouragement strategies may change over the course of sustained interaction, and that this process may differ depending on the conversational partner. Theoretical accounts of encouragement effectiveness should therefore consider not only which strategy is evaluated as effective, but also how such evaluations develop over sustained interaction and whether this process differs by conversational partner.

Second, the findings suggest that support matching in HRI may operate at multiple levels. The Optimal Matching Model emphasizes the match between support type and stressor characteristics (Cutrona and Russell, 1990), and the present findings suggest that additional forms of matching may be relevant in sustained HRI. Users may consider not only whether a strategy is appropriate for a particular worry, but also whether the specific content fits their preferences and whether the robot is perceived as capable of providing that form of support. Perceived effectiveness of robot-delivered encouragement may therefore involve at least three levels of matching: strategy-worry fit, content-user fit, and support-partner capability fit. Selecting a strategy solely on the basis of worry type may therefore be insufficient for effective support in sustained interaction.

Third, the CASA paradigm suggests that people respond socially to technology (Nass et al., 1994; Nass and Moon, 2000), and participants in this study similarly disclosed personal worries to the robot and reported closeness, trust, and being listened to. However, when evaluating encouragement such as offering specific actions, participants did not simply accept robot-delivered support in the same way as human-delivered support. They also judged whether the robot could realistically provide the help it offered, and whether a given response was appropriate coming from a robot rather than a human partner (Finding 6). Responding socially to a robot therefore does not imply evaluating its support by the same standards applied to human support. Theoretical accounts of supportive HRI should consider social responsiveness, perceived capability, and partner-role expectations as concurrent components of the evaluation process.

This study also offers several practical design considerations for encouragement functions in conversational robots for older adults.

First, encouragement effectiveness depends on matching at two levels: (1) strategy type to worry type (systems should identify worry nature and adjust accordingly), and (2) strategy content to individual preferences (misalignment with daily habits and personal preferences may lead to substantially lower perceived effectiveness). This suggests that systems should establish user preference profiles to enable personalized matching at both levels. Systems should also identify the type of support the user currently wants, such as advice, listening, assistance, or temporary distraction, rather than selecting a strategy solely on the basis of worry type.

Second, perceived effectiveness is modulated by users’ subjective sense of novelty. Even with objectively different content, if users perceive suggestions as similar in kind (e.g., variations of “have something warm to drink” like hot tea or warm soup, or “look up some information” like hospital resources or recipes across weeks), perceived effectiveness may decline. This suggests that system design should avoid not only literal repetition but also perceived similarity, tracking suggestion type history to ensure content feels sufficiently varied.

Third, this study highlights the design value of being listened to. Most participants lacked suitable outlets for everyday disclosure, and being listened to may carry independent support value beyond encouragement quality. Thus, system design should not prioritize high-quality advice alone, but also sustained responsiveness and memory continuity for what users have shared. The safe conversational space created by the interaction itself may be an important source of long-term emotional support effects.

Fourth, this study addresses the alignment of robot verbal content with capability signals from its physical appearance. Although this study’s encouragement design was intentionally constrained to RoBoHoN’s actual functional capabilities, such as looking up information or setting reminders, some participants still perceived a capability mismatch for physical worries. This suggests that capability alignment depends not only on objective system functions but also on the robot’s physical appearance and users’ prior intuitive judgments. Encouragement utterances should therefore avoid not only commitments exceeding actual capabilities, but also expressions evoking physical action or real-world execution. For small, childlike, or pet-like robots, verbal content may emphasize companionship, reminders, information assistance, and emotional support.

Finally, embodied feedback should be coordinated with verbal encouragement. Participants’ responses to RoBoHoN’s illuminated-eye feedback suggest that even simple nonverbal cues can strengthen the impression that the robot is listening (Finding 4). Gaze, lighting, posture, and turn-taking feedback should therefore not be designed separately from verbal responses, but should work together to create a coherent supportive interaction.

Given the small sample and descriptive analyses, these implications should be regarded as preliminary.

### 5.3 Limitations and future directions

This study has several limitations that should be considered when interpreting the findings, each suggesting directions for future research.

First, the sample size was small, with only four to five participants per group, and the results were based primarily on descriptive analyses that do not support robust statistical inference. The findings should therefore be regarded as preliminary trends. Furthermore, the two human partners were unevenly assigned (7:1) and both female, and only one robot model (RoBoHoN) was used, limiting generalizability. Future research should replicate these findings with larger samples, more diverse partners, and a broader range of robot types.

Second, this study did not include a no-encouragement control condition, making it impossible to fully disentangle specific encouragement effects from the conversational intervention itself. Future research could incorporate a listening-only control condition to clarify the relative contributions of being listened to versus being encouraged in shaping changes in SWB.

Third, all participants were older Japanese adults, most in relatively good health and with some social activity engagement. The observed disclosure barriers may be culturally specific, and whether findings generalize to other cultural backgrounds or higher-risk groups (e.g., greater social isolation or poorer health) remains to be examined. Future research should include participants from diverse cultural backgrounds and varying health and social isolation levels to directly examine the moderating roles of cultural norms and individual characteristics.

Fourth, the preliminary survey used an online scenario-based design with no physical robot or visual stimuli, creating an ecological validity gap relative to the main experiment. In addition, the age range differed between the preliminary survey and main experiment, which may have affected generalizability of the strategy selection. Future surveys could incorporate robot photographs, videos, or simulated dialogue to more accurately predict strategy effectiveness in real interactions.

Fifth, although the robot’s voice was adjusted in pitch and speech rate to approximate the human partners’ vocal characteristics, it may not have fully matched RoBoHoN’s physical appearance. In addition, the inevitable delays between speech recognition and response generation introduced systematic differences in conversational timing between the robot and human conditions. Future research could explore more naturalistic voice synthesis solutions and systematically record conversational timing parameters to improve comparability across conditions.

Sixth, human partners naturally used body language and non-verbal cues during interaction, whereas RoBoHoN’s physical movements were disabled, with only the eye LED display retained. This created a systematic asymmetry in non-verbal communication between the two conditions. Future research could explore selectively enabling robot non-verbal behaviors while maintaining verbal strategy control to assess the independent contribution of embodiment cues to encouragement effectiveness.

Seventh, the study was conducted in a community center with weekly short sessions, which may not reflect natural long-term robot use at home. Moreover, the study did not account for participants’ baseline SWB levels, which may influence the interpretation of SWB changes. Some baseline scores were close to the upper limit of the scale, suggesting potential ceiling effects that may have limited the sensitivity to detect intervention-related improvements. Although diary data tracked external life events, SWB may have been influenced by other factors (e.g., family, health, community activities) that could not be fully accounted for. Future research could be conducted in home settings and incorporate objective measures (e.g., physiological indicators, behavioral logs, long-term usage data) to more comprehensively examine the longitudinal effects associated with the intervention.

Eighth, the prompting framework was predefined. Although the dialogue context was updated with each participant response, the system prompt and objective templates for each turn were not adapted based on the participant’s state or through any learning algorithm. Future research could investigate prompting methods based on reinforcement learning or other adaptive approaches that adjust the robot’s interaction style in response to the user’s affective or conversational state. Such research could examine whether this adaptation enhances affective engagement and strengthens the effects of encouragement during sustained interaction.

Finally, this study covered only a 6-week period and compared just two encouragement strategy types. Whether novelty effects might emerge over extended interaction periods, whether the affective gains observed in the robot condition can be sustained over time, and the effects that other encouragement strategy types might produce all remain open questions. Future research should therefore lengthen the intervention timeline and also directly measure mechanism variables such as perceived agency, social presence, sense of being heard, and perceived capability match, thereby enabling future studies to quantitatively examine the possible psychological mechanisms underlying the associations between different encouragement strategies and SWB.

### 5.4 Conclusion

This exploratory 6-week longitudinal study directly compared high- and low-perceived-efficacy encouragement strategies delivered by a human partner versus a social robot in relation to the subjective well-being of older Japanese adults. A preliminary survey identified offering specific actions as a high-efficacy strategy and distraction as a low-efficacy strategy. These two strategies were then implemented in the 6-week main experiment (human partner vs. social robot × high-vs. low-efficacy). The findings suggest that encouragement may improve older adults’ SWB, and that the pattern of improvement may vary by conversational partner and SWB dimension. Quantitative data showed that, across all four conditions, mean AWB and CWB scores were higher at Week 6 than at the pre-intervention assessment. In the human condition, high-efficacy encouragement appeared to show a larger increase in CWB than low-efficacy encouragement; in the robot condition, high-efficacy encouragement appeared to show a larger increase in AWB than low-efficacy encouragement. Perceived encouragement effectiveness also appeared to change over the course of sustained interaction, with mean ratings across all four conditions higher at Week 6 than at Week 3, and the degree of differentiation between the two strategies appeared to differ by conversational partner: they remained clearly separated in the human condition, whereas by Week 6 both received similarly high mean ratings in the robot condition. Qualitative data further suggested that, within the Japanese cultural context of this study, the conversational space itself may provide supportive value in addition to the specific content of the encouragement strategy delivered.

Taken together, the findings raise the possibility that the same encouragement strategy may operate differently depending on who delivers it, both in which dimension of well-being it is associated with and in how clearly it is distinguished from alternative strategies. On this basis, social robots designed for emotional support among older adults may be better understood not simply as substitutes for human supporters, but as complementary resources capable of providing a low-threshold, sustained outlet for everyday disclosure. Although the findings remain preliminary because of the small sample and descriptive analyses, this study provides early longitudinal insight into encouragement over sustained interaction in HRI contexts and offers design implications for emotionally supportive robots for older adults.

## Data Availability

The original contributions presented in the study are included in the article/Supplementary Material, further inquiries can be directed to the corresponding authors.
