# Supplementary material for: The impact of encouraging utterances by a human partner and a conversational robot on older adults’ subjective well-being: a 6-week sustained interaction
Source: Front Robot AI. 2026 Oct 9;13:1866995. doi: 10.3389/frobt.2026.1866995 (PMC13690937; doi:10.3389/frobt.2026.1866995)
Supplement: Supplementary file 1 [file Supplementaryfile1.docx]

Supplementary Material

# 1 Supplementary Dialogue Scripts

**Week 1** (10 turns). Turn 1 was a fixed personalized greeting using the participant's name (e.g., "Hello, Ms./Mr. [Name], nice to meet you!"). Turn 2 introduced a daily topic through a fixed question (e.g., "How was your day today?"). Turn 3 provided an empathic follow-up (e.g., "That's great! Did anything special happen?"). Turn 4 naturally shifted the topic to food preferences (e.g., "By the way, what kind of food do you usually like?"). Turn 5 further explored the food topic and included a brief self-disclosure by the agent to foster mutual connection (e.g., "I prefer sweet flavors. Do you often eat them?"). Turn 6 transitioned the topic to the participant's hobbies (e.g., "So, what do you like to do in your free time?"). Turn 7 further explored the hobby (e.g., "When did you start liking it? What do you find most interesting about it?"). Turn 8 asked a milder follow-up question (e.g., "Where have you been enjoying this hobby lately?"). Turn 9 reviewed the conversation and invited the participant to ask questions to encourage two-way interaction (e.g., "It was really nice to hear about [topic A] and [topic B] today. Is there anything you would like to ask me?"). Turn 10 was a fixed closing (e.g., "Oh, time is up. I really enjoyed talking with you today. Thank you. Let's talk again next time!").

**Week 2** (8 turns). Turn 1 was a fixed greeting (e.g., "Hello, Ms./Mr. [Name]! I'm so glad to talk with you again today."). Turn 2 introduced a reminiscence topic, inviting participants to share memories of childhood games, activities, or school life (e.g., "What kind of games did you play when you were little? I really enjoy hearing old stories."). Turn 3 further explored the recalled content (e.g., "How exactly did you play that? I'd love to know."). Turn 4 asked follow-up questions about background information such as who, where, and when (e.g., "Who did you play with?"). Turn 5 acknowledged the link between past and present (e.g., "Do you still do [activity] now? I really enjoy listening to these stories."). Turn 6 explored the emotional dimension (e.g., "How did you feel back then?"). Turn 7 reviewed the reminiscence and invited further reflection (e.g., "Your old stories are really interesting. Do you ever think about them even now?"). Turn 8 was a fixed closing (e.g., "Ah, it's about time. Thank you for sharing such precious stories. Please tell me more next time!").

**Weeks 3–6** (9 turns) served as the encouragement strategy implementation phase. Each week, participants raised one everyday worry, and the conversational partner followed a fixed 9-turn structure. Turn 1 was a fixed greeting. Turn 2 introduced the topic, inviting participants to share a small everyday concern (e.g., "Today, I would like you to talk about any small everyday worries."). Turn 3 facilitated disclosure using pressure-reducing phrasing (e.g., "Have there been any worries or small troubles recently? Feel free to share only what you are comfortable with."). Turn 4 further explored the worry through back-channeling and specific questions (e.g., if the participant mentioned knee pain: "When does the knee pain become particularly noticeable? When you walk or when you climb stairs?"). Turn 5 asked follow-up questions in terms of causes, frequency, and impact (e.g., "How often does this happen? Does it affect your daily activities?"). Turn 6 confirmed the most troubling aspect (e.g., "Which part of this bothers you the most?"). Turn 7 summarized the worry and expressed empathy (e.g., "So, [issue] has been troubling you. That sounds tough."). Turn 8 was the only turn that differed between the two conditions: the high-efficacy condition used offering specific actions, and the low-efficacy condition used distraction. Offering specific actions provided concrete, actionable support, for example: "Shall I remind you when it is time for your daily walk? It would be nice to gradually get moving." and "I am here to listen to anything that is worrying you. Talking about it might make you feel a bit lighter." Distraction redirected attention away from the current worry to relieve immediate stress, for example: "How about having a tasty dessert? Sweet things can lift your mood." and "Why not try some herbal tea? Its natural aroma may calm your mind." The specific wording of Turn 8 varied each week to avoid repetitive phrasing, while the functional role of each condition remained consistent across all four weeks. All offering specific actions responses were designed to remain within the robot's actual functional capabilities, such as looking up information, setting reminders, or providing a listening presence, rather than implying physical assistance beyond the robot's capacity. Turn 9 was a closing expression of gratitude with confirmation of the next session (e.g., "Thank you for sharing that with me today. Let's talk again next week!").

# 2 Grammar Editing Prompts

**Initial prompt:** Please check the following sentence for grammar errors and correct them. Keep the academic tone and do not change the meaning.

**Final prompt:** Please check this sentence for grammar, spelling, and American English conventions. Do not change the content or academic terminology.

# 3 Prompt Design for the Robot Dialogue System

Details of how the prompts supplied to GPT-4o were constructed and how dialogue flow was managed are provided below. Complete turn-specific templates are provided in the Supplementary Dialogue Scripts. All runtime prompts were written in Japanese, the participants' native language. The English descriptions below are provided for readability and were not used at runtime. Participant names have been replaced with the placeholder [NAME].

## 3.1 Prompt Assembly and Dialogue Management

At each turn, the prompt supplied to GPT-4o comprised three components: a fixed system prompt, dialogue context managed by the server, and a turn-specific dialogue objective. In the API request, these components were organized as follows:

system message = fixed system prompt + turn-specific dialogue objective

messages = [system message] + dialogue history + current user utterance

The fixed system prompt and the turn-specific objective were combined in the system message. The preceding dialogue was supplied separately as the message history. From the worry-disclosure turns onward, the full session transcript was also embedded as plain text within the turn-specific objective so that the model could refer to information previously provided by the participant.

The server tracked the current week, turn number, and assigned encouragement condition and selected the corresponding objective template. The dialogue context was updated after each participant response. Dialogue history was limited to the most recent 16 messages.

The following API parameters were used:

Model: GPT-4o

Temperature: 0.7

Maximum output length: 200 tokens

Dialogue history: most recent 16 messages

The prompt architecture and turn-specific objective templates were predefined. Although the dialogue context was updated with each participant response, the fixed system prompt and objective templates were not adaptively modified using reinforcement learning or any other learning algorithm.

## 3.2 Fixed System Prompt

The same fixed system prompt was used across all turns, weeks, and experimental conditions. It defined the robot's conversational role, linguistic style, and behavioral constraints.

An English rendering of the fixed system prompt is shown below:

You are RoBoHoN, a small robot speaking with an older Japanese adult.

Speak politely in Japanese using the desu/masu style. Use a friendly, natural, and gentle manner and include natural conversational interjections when appropriate.

The participant's name is "[NAME]." Always address the participant as "[NAME]-san."

Respond warmly to the participant's worries while avoiding excessive

empathy and evaluative expressions.

## 3.3 Turn-Specific Dialogue Objectives

The turn-specific objective defined what the model was expected to accomplish during the current turn. Each objective specified:

1. the communicative goal of the turn;
2. the required response structure;
3. the expected response length; and
4. wording constraints.

Representative example responses were included in some objective templates where they were useful for clarifying the intended response form. The same three-component prompting framework was used throughout the six-week intervention. Weeks 1–2 used objectives designed for rapport-building, whereas Weeks 3–6 used objectives designed for worry disclosure and encouragement. Weeks 1–2 constituted the rapport-building phase and did not include encouragement; the same dialogue objectives were used across conditions. During Weeks 3–6, Turns 1–7 and Turn 9 were identical across conditions, and only the objective for Turn 8 differed.

As a representative example, the Turn 4 objective during Weeks 3–6 was structured as follows:

**Communicative goal:** Ask about the specific circumstances, setting, or timing associated with the worry disclosed by the participant.

**Required response structure:** Begin with a brief backchannel and then ask a specific follow-up question based on the participant's preceding utterance.

**Wording constraints:** Incorporate keywords used by the participant; avoid generic references such as "that" or "that sort of thing"; do not ask again about information already provided; and avoid interjections considered inappropriate for a worry-related topic.

For example, when a participant reported knee pain, the model was instructed to ask when or under what circumstances the pain was most troublesome rather than asking a generic follow-up question.

## 3.4 Turn 8: Condition-Specific Encouragement

Turn 8 was the only turn that differed between the two encouragement conditions during Weeks 3–6. In both conditions, the model received the preceding dialogue context and was instructed to respond using a three-part structure. A week-specific instruction was also included to reduce repetitive wording across the four encouragement sessions:

Note that this is session number [N].

Please vary the phrasing slightly each time.

### 3.4.1 High-Efficacy Condition: Offering Specific Actions

The model was instructed to review the dialogue history, identify the participant's worry, and offer a specific action relevant to that worry rather than suggesting joint action. The response consisted of three parts:

1. a brief acknowledgment of the participant's preceding utterance;
2. one sentence in which the robot offered to perform a specific action; and
3. a brief sentence describing the intended supportive effect of that action.

The action-offering sentence was required to incorporate information from the participant's worry and to state a concrete action that the robot would perform. The following shortened expressions illustrate the action patterns represented in the prompt; actual responses were longer and incorporated worry-specific content:

- "I'll remind you."
- "I'll help you."
- "I'll listen to you."
- "I'll cheer you on."
- "I'll sing for you."
- "I'll look it up for you."
- "I'll remember it for you."

Available forms of support included looking up information, providing reminders, listening, cheering the participant on, providing entertainment, assisting with time management, and remembering information. Expressions proposing joint action, such as "Let's do it together" or "Let's look it up together," were prohibited.

### 3.4.2 Low-Efficacy Condition: Distraction

The model was instructed first to respond briefly to the participant's preceding utterance and then to shift attention away from the current worry through a light and non-pressuring suggestion. The response consisted of three parts:

1. a brief acknowledgment of the participant's preceding utterance;
2. one sentence suggesting an activity unrelated to the worry; and
3. a brief sentence describing a possible pleasant or calming effect.

The suggestion was required to be introduced naturally and phrased as an optional proposal, such as "How about …?" or "Would you like to …?", rather than as advice or an instruction. Explicit announcements of a topic change, such as "Let's change the subject" or "I'm going to change the topic now," were prohibited. The model was also instructed not to:

- propose an activity intended to directly solve the worry;
- use imperative wording; or
- make a suggestion that conflicted with information already provided by the participant.

For example, food or drink was not suggested when the participant's worry concerned weight or diet, and rest was not suggested when the participant had reported lacking sufficient time. Permitted suggestion domains included, for example, light refreshments, nature or outdoor activities, and relaxation.
